# Cinnamaldehyde mitigates MASLD through SIRT1/FOXO1-induced autophagy and synergistic gut microbiota modulation

**DOI:** 10.1038/s41538-026-00815-6

**Published:** 2026-04-09

**Authors:** Xiaoran Wang, Yagang Song, Wenyu Zhao, Yuting Liu, Yiping Fu, Yu Zhang, Quanyou Zhao, Mingsan Miao, Wenxia Zhao, Xianbo Wang, Zhanzhan Li

**Affiliations:** 1 Department of Digestive Diseases, The First Affiliated Hospital of Henan University of Chinese Medicine, Zhengzhou, China; 2 First Clinical Medical College, Henan University of Chinese Medicine, Zhengzhou, China; 3 Academy of Chinese Medicine Sciences, Henan University of Chinese Medicine, Zhengzhou, China; 4 School of Rehabilitation Sciences, Henan University of Chinese Medicine, Zhengzhou, China; 5 Pharmacy College, Henan University of Chinese Medicine, Zhengzhou, China; 6 Center for Integrative Medicine, Beijing Ditan Hospital, Capital Medical University, Beijing, China

**Keywords:** Diseases, Gastroenterology, Microbiology

## Abstract

Metabolic dysfunction-associated steatotic liver disease (MASLD) is a global health burden with limited therapeutic options. *Cinnamomum cassia*, a medicinal-food homologous plant, contains principal bioactive cinnamaldehyde (CA), whose anti-MASLD mechanisms require clarification. ‌This study employed both a high-fat diet (HFD)-induced MASLD model and a free fatty acid (FFA)-stimulated cell model. CA administration attenuated intracellular lipid accumulation in vitro and ameliorated both hepatic steatosis and systemic hyperlipidemia in vivo, while inhibiting hepatic lipid peroxidation. Mechanistically, integrated RNA-seq, network pharmacology, siRNA, immunofluorescence, and transmission electron microscopy analyses identified the SIRT1/FOXO1–autophagy axis as CA’s key regulatory pathway. Gut microbiome profiling revealed CA’s capacity to ameliorate HFD-induced dysbiosis, particularly enriching *Lachnospiraceae_NK4A136*. Fecal microbiota transplantation (FMT) and Spearman correlations link serum lipids and hepatic injury factors to gut microbiota, indicating partially microbiota-mediated metabolic modulation by CA. Collectively, CA ameliorates MASLD through coordinated autophagy enhancement and microbial homeostasis restoration, holding promise as a functional food ingredient for ‌metabolic liver disease prevention.

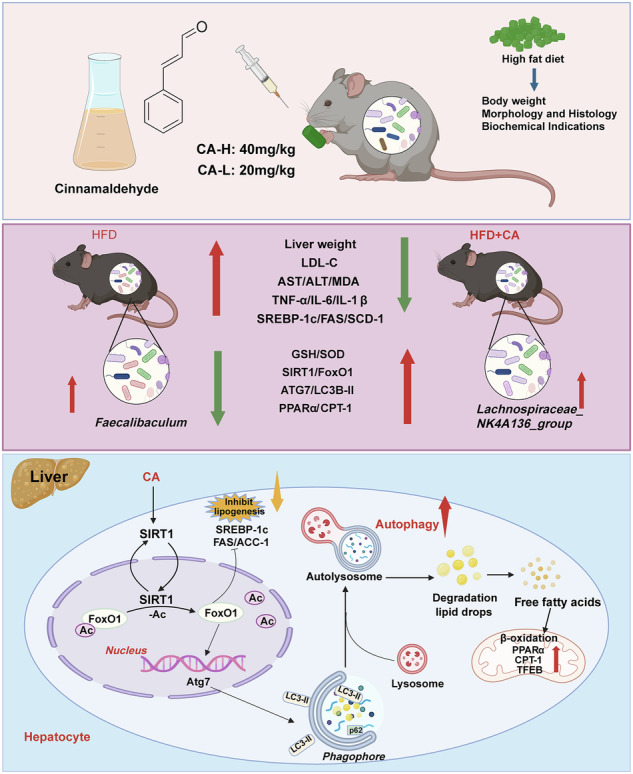

## Introduction

MASLD is a persistent metabolic disorder associated with stress-induced liver damage, pathologically manifested as triacylglycerol accumulation within hepatocytes, leading to macrovesicular or microvesicular steatosis^1,2^. Progressive lipotoxicity triggers hepatocyte apoptosis and hepatic fibrosis, potentially causing cirrhosis or hepatocellular carcinoma^3,4^. Currently, Resmetirom, a thyroid hormone receptor β agonist, represents the first FDA-approved therapy for metabolic dysfunction-associated steatohepatitis (MASH). Nevertheless, its high cost and adverse effects, such as nausea, vomiting, and diarrhea, limit its broad clinical application^5^. Phytochemicals and plant-sourced foods emerge as promising therapeutic interventions for metabolic diseases, given their pleiotropic bioactivity and toxicological advantages^6,7^. Notably, these compounds represent a frontier in translational MASLD research as non-pharmacological candidates. Nevertheless, current agents used in MASLD, such as curcumin, polyene phosphatidylcholine, ursodeoxycholic acid, and silymarin—still show suboptimal efficacy^8^. CA, a natural aldehyde extracted from *Cinnamomum cassia* bark, is an FDA-approved food additive^9–11^. Recently, dietary CA supplementation has been proposed to modulate hepatic lipid metabolism and reduce lipid accumulation, though its mechanisms against MASLD remain poorly understood^12,13^.

Autophagy, an essential cellular homeostasis mechanism involving degradation of damaged organelles and misfolded proteins, is critically implicated in MASLD pathogenesis^14,15^. Research has indicated that the hepatic autophagic activity is diminished in MASLD patients, resulting in pathological lipid accumulation^16^. Impaired autophagy may lead to the excessive secretion of inflammatory mediators, accelerating the progression of MASLD to MASH^17^. SIRT1 is an NAD+-dependent deacetylase that modulates the function of numerous transcription factors and proteins, including FOXO1, through deacetylation^18^. SIRT1 activates FOXO1 through deacetylation, promoting its nuclear translocation to induce autophagy^19^. Concurrently, activated FOXO1 suppresses lipogenesis by downregulating key enzymes, including acetyl-CoA carboxylase-1 (ACC-1) and fatty acid synthase (FAS). The SIRT1/FOXO1 signaling axis significantly inhibits the pathogenesis and progression of MASLD by inducing autophagy, regulating lipid metabolism, mitochondrial function, and inflammatory response^20^.

In this study, we investigated the therapeutic efficacy of CA against MASLD and delineated its mechanistic basis. MASLD models were induced in high-fat diet (HFD)-fed mice and free fatty acid (FFA)-exposed HepG2 cells. Integrated RNA-Seq and network pharmacology revealed that CA’s activity was enriched in the SIRT1 and FOXO1 signaling pathways. The involvement of SIRT1/FOXO1-mediated hepatic autophagy in the lipid-reducing effects of CA was verified using scanning electron microscopy, immunofluorescence, inhibitor reversal assays with 3-methyladenine (3-MA) or nicotinamide (NAM), and a Foxo1 knockdown HepG2 cell line. Furthermore, 16S rRNA sequencing analyzed CA-modulated gut microbiota changes in MASLD mice, and functional studies evaluated microbial roles in ameliorating MASLD. These findings offer new perspectives on CA-induced autophagy activation and its potential utility in MASLD treatment.

## Results

### CA alleviates HFD-induced hepatic steatosis in mice

**Fig. 1:**
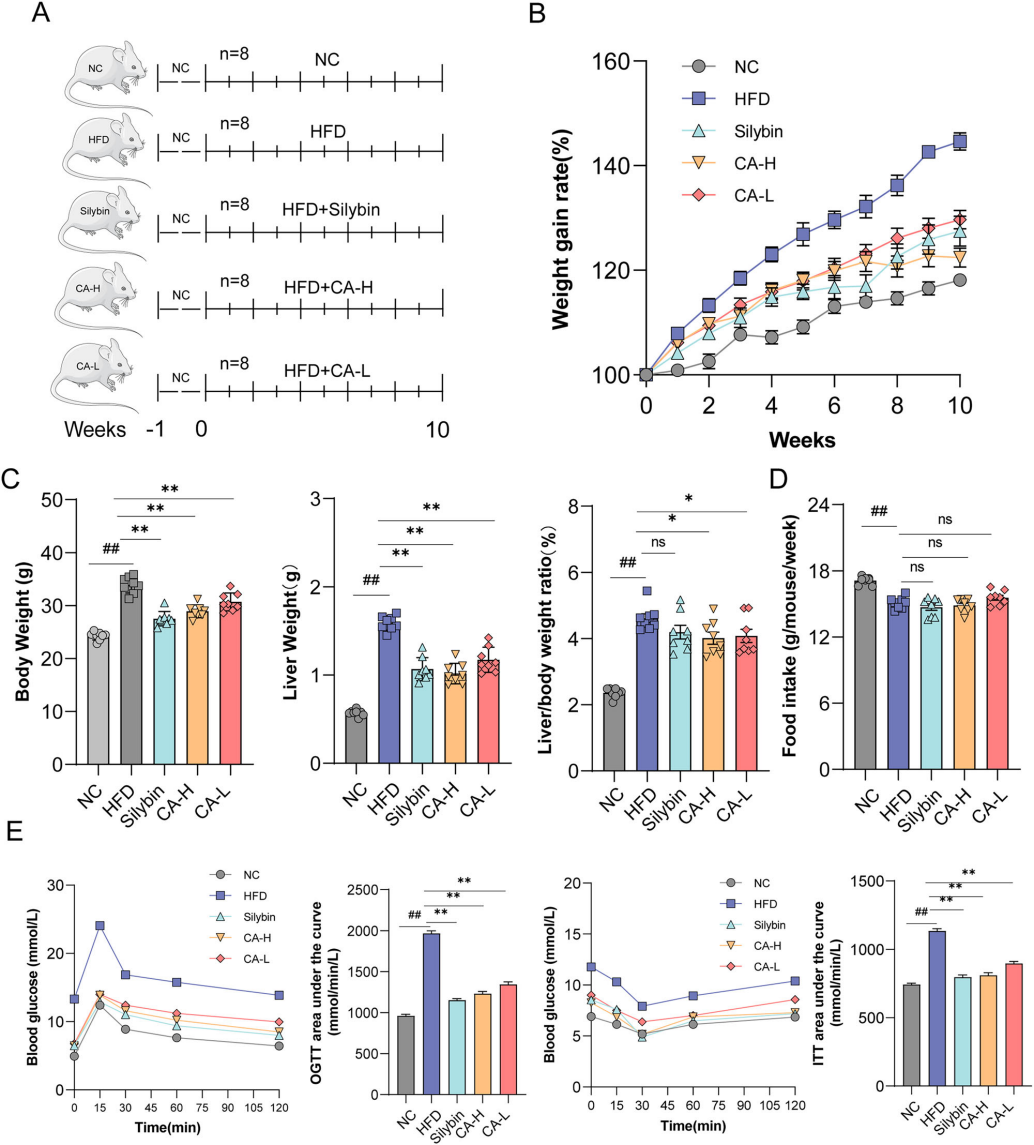
Effects of CA on appearance indices in HFD-fed mice. **A** Experimental outline. **B** Grams of weight gain rate measured over time. **C** Liver weight and ratio of liver weight to body weight (LW/BW). **D** Food intake was measured once a week. **E** OGTT and ITT. Data are shown as the means ± SEMs (*n* = 8). Compared with the NC group, ^#^*P* < 0.05, ^##^*P* < 0.01; compared with the HFD group, *P < 0.05, **P < 0.01.

MASLD is caused by impaired lipid metabolism, characterized by excessive hepatic triglyceride accumulation, which can be induced by a persistent HFD. To assess the effects of CA on MASLD progression, mice were maintained on a high-fat diet with daily oral CA administration for 10 weeks (Fig. 1A). Compared with normal mice in the NC group, HFD induced typical pathological features of MASLD, including liver index, body fat accumulation, and significant weight gain. Strikingly, both doses of CA treatment attenuated body weight gain, liver weight, and liver index in HFD-fed mice (Fig. 1B,C). However, no significant differences in food intake were observed among groups (Fig. 1D), indicating its safety for long-term use. Furthermore, the OGTT and ITT results demonstrated that the glucose regulation capacity of the HFD group was impaired, with a significantly higher AUC compared to both the high-dose and low-dose CA groups (*P*-value < 0.01), which suggested that CA intervention markedly enhances glucose regulation and insulin sensitivity in HFD-fed mice (Fig. 1E).

**Fig. 2:**
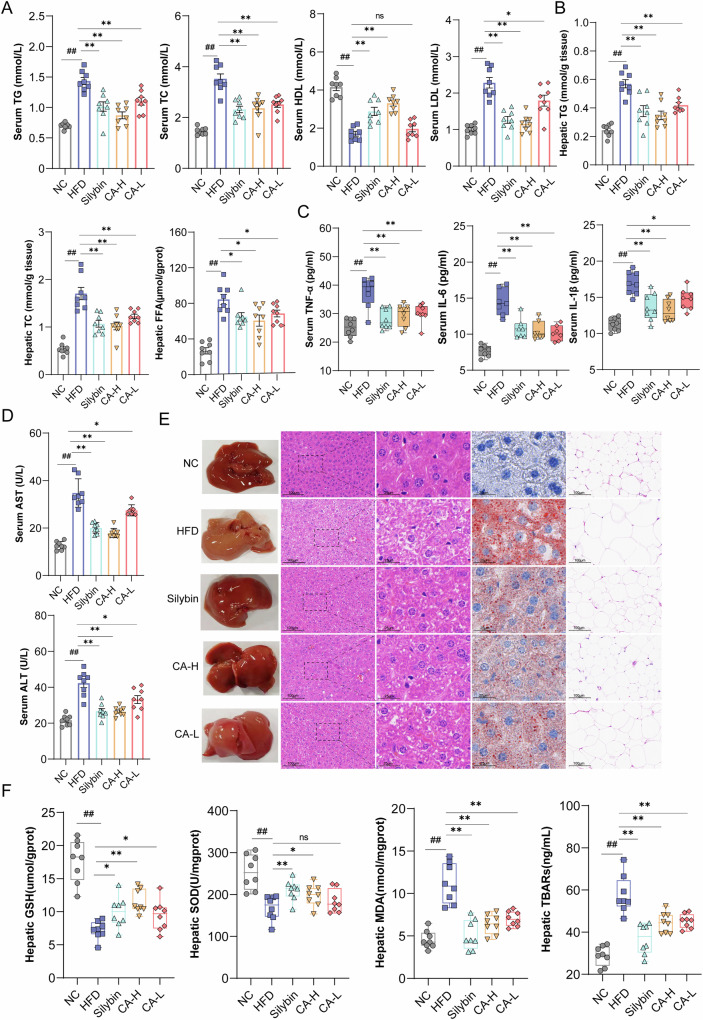
CA reduces lipid accumulation, inflammatory factors, liver injury factors, and oxidative stress levels in HFD-fed mice. **A** Serum TG, TC, HDL, and LDL. **B** Hepatic TG, TC, and FFA. **C** Serum TNF-α, IL-6, and IL-1β. **D** Serum AST and ALT. **E** Representative images of liver pictures, H&E staining of liver and epididymis fat sections, and Oil-red O staining of liver sections. Scale bars: 100 and 25 μm. **F** Hepatic GSH, SOD, MDA, and TBARs. Data are shown as the means ± SEMs (*n* = 8). Compared with the NC group, ^#^*P* < 0.05, ^##^*P* < 0.01; compared with the HFD group, *P < 0.05, **P < 0.01.

Chronic fat accumulation in hepatocytes induces hepatic steatosis, promoting oxidative stress and inflammation^21^. CA significantly attenuated HFD-induced steatosis, demonstrated by decreased hepatic TG, total TC, and FFA, and reduced AST, ALT, TG, and TC levels in serum. In addition, only the high dose of CA can significantly increase HDL content; both doses can suppress serum LDL (Fig. 2A, B, and D). Moreover, the reversal of pro-inflammatory cytokines TNF-α, IL-6, and IL-1β, as confirmed by RT-qPCR analysis showing their significant reduction in liver tissue of the CA-treated group compared to the model group, suggests that CA has the potential to ameliorate hepatic inflammation(Figs. 2C and S1A). Consistent with these findings, H&E and ORO staining revealed that the pathological lesion of massive accumulation of lipid droplets (LDs) in the liver, distorted hepatic lobules, inflammatory cell infiltration, and ballooning caused by HFD feeding can be alleviated by CA intervention, and the high-dose group showed a better effect (Fig. 2E). The NAS score results indicated significant steatohepatitis activity in the model group, which was markedly reduced following high-dose CA intervention (Fig. S1B). In addition, Masson’s staining showed minimal overall collagen deposition across groups. The model group exhibited a mild increase in collagen fibers, whereas the positive control and both high- and low-dose CA groups showed less blue-stained collagen, indicating that this model represents an early stage of MASLD progression (Fig. S1C).

Oxidative stress-induced inflammatory response, primarily characterized by excessive production of peroxides resulting from the disruption of oxidant–antioxidant homeostasis, represents a crucial pathogenic mechanism in hepatic fibrogenesis^22^. Both low and high doses of CA demonstrated significant hepatoprotective effects through upregulation of endogenous antioxidant defense systems, as evidenced by elevated GSH levels and reduced MDA and TBARs concentrations. Notably, the high-dose CA intervention exhibited additional antioxidant efficacy by enhancing SOD activity (Fig. 2F). Taken together, these results indicate that CA can ameliorate the progression of diet-induced MASLD and its associated metabolic disorders. In addition to the primary focus on CA’s effects, we also evaluated Silybin’s impact on hepatic steatosis and inflammation for comparative purposes. While both compounds reduced lipid accumulation, CA demonstrated superior efficacy compared to Silybin.

### SIRT1/FOXO1 signaling pathway mediates the therapeutic mechanism of CA in MASLD

**Fig. 3:**
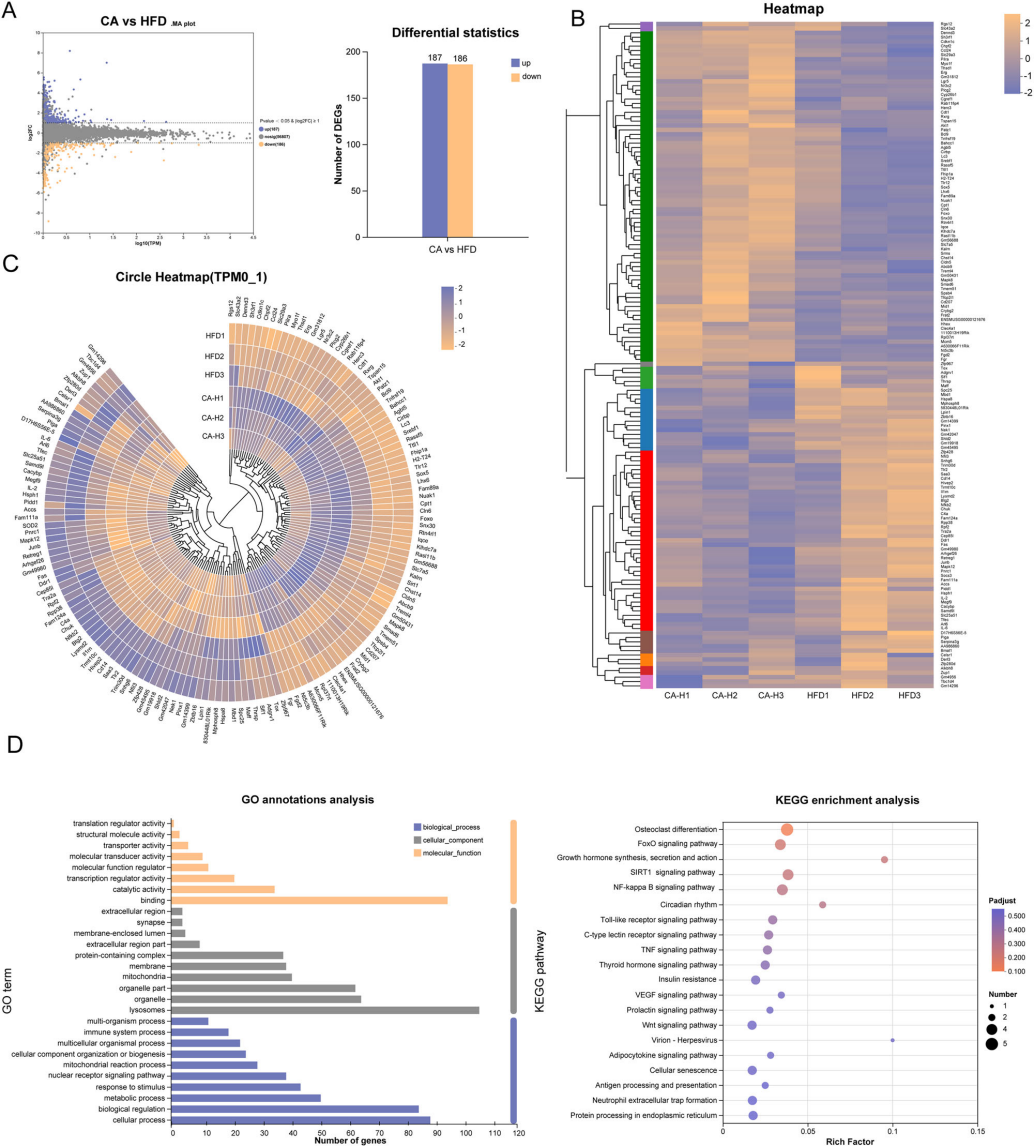
Liver transcriptome analysis in CA-treated HFD-fed mice. **A** Volcano plot showing differentially expressed genes between normal samples and CA samples, with blue dots representing upregulated genes and yellow dots representing downregulated genes. **B** Heatmap of cluster analysis, where yellow indicates high gene expression, and blue indicates low gene expression. **C** Heatmap, where blue indicates high gene expression, and yellow indicates low gene expression. **D** Gene Ontology (GO) and Kyoto Encyclopedia of Genes and Genomes (KEGG)-enrichment analysis.

To investigate CA-associated transcriptional changes and mechanisms, we performed unbiased RNA sequencing and conducted gene set enrichment analysis (GSEA) to characterize functionally enriched gene sets exhibiting significant differential expression between control and CA samples. GSEA identified 373 genes with the most significant differential expression (FDR < 0.05, |log2FC| > 1), comprising 187 upregulated and 186 downregulated genes (Fig. 3A). Heatmap analysis revealed significant enrichment of differentially expressed genes regulating lipid metabolism (e.g., Accs, Fas, and Cpt-1), inflammatory response (e.g., IL-6 and IL-2), and autophagy (e.g., Akt1, Sirt1, and LC3) (Fig. 3B,C). GO term analysis revealed that differentially expressed genes were associated with metabolic processes, mitochondrial reaction processes, and nuclear receptor signaling pathways. Notably, the KEGG pathway analysis revealed significant enrichment of differentially expressed genes in pathways including the “FoxO signaling pathway”, “NF-kappa B signaling pathway”, and “SIRT1 signaling pathway” (Fig. 3D).

**Fig. 4:**
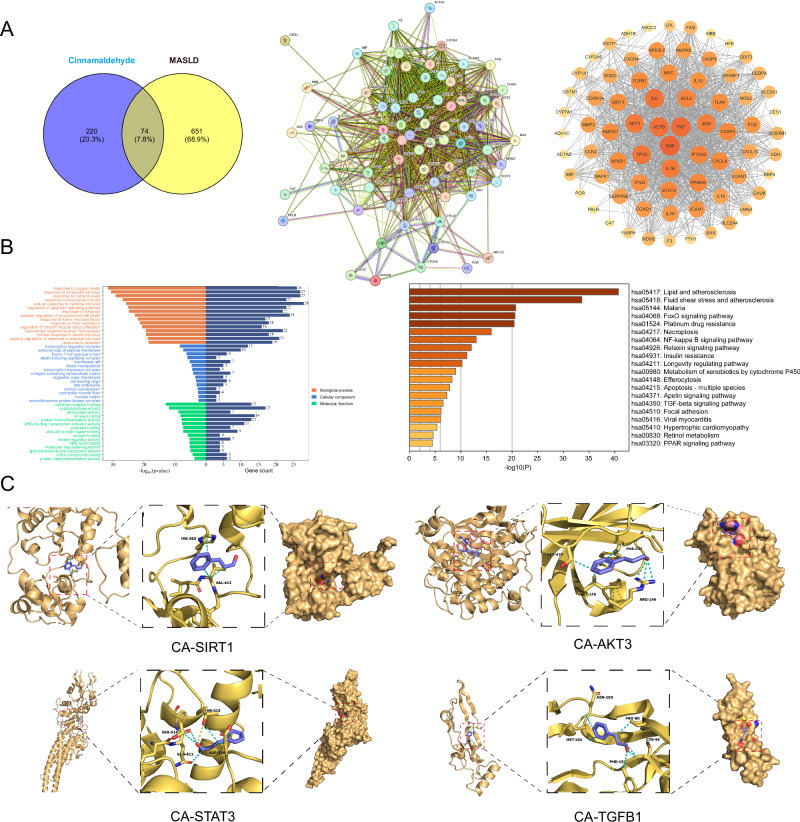
Network pharmacology analysis of CA intervention in MASLD. **A** Common targets between CA and MASLD and protein–protein interaction network. **B** GO and KEGG enrichment analysis. **C** Molecular docking mode of CA with SIRT1, AKT3, STAT3, and TGFB1.

To elucidate the core therapeutic targets of CA in MASLD management, we implemented an integrated computational approach combining network pharmacology analysis and molecular docking simulations. As illustrated in Fig. 4A, Venn diagram analysis revealed 74 overlapping targets between CA and MASLD, with ALB, TNF, ACCs, IL-6, TP53, SIRT1, and AKT1 emerging as the principal anti-MASLD targets of CA. GO and KEGG pathways demonstrated significant clustering of these targets in biological processes, including “cellular response to oxygen-containing compound”, “regulation of response to cytokine stimulus”, and “FoxO signaling pathway transduction” (Fig. 4B). SIRT1, an NAD+-dependent deacetylase, serves as a master regulator of autophagy by modulating autophagic activity through deacetylation of multiple autophagy-related proteins^23,24^. FOXO1, an evolutionarily conserved transcription factor, governs the transcriptional network of autophagy-related genes and serves as a critical mediator in cellular adaptive responses to metabolic stress^25^. The SIRT1/FOXO1-signaling pathway represents a crucial regulatory mechanism in cellular autophagy^26^. Transcriptomic analysis also revealed that CA intervention significantly upregulated the expression of LC3, SIRT1, and FOXO1 genes.

Furthermore, we evaluated the potential of CA to modulate the SIRT1 pathway through molecular docking, focusing on its interactions with SIRT1, AKT1, STAT3, and TGFB1, in which AKT1, STAT3, and TGFB are known to regulate SIRT1 expression or function. As illustrated in Fig. 4C, the ligand is positioned within the active pocket of the protein, forming multiple hydrogen bonds and hydrophobic interactions with key residues. Specifically, CA was predicted to establish hydrogen bonds with HIS (363) and VAL (412) of SIRT1. In AKT3, hydrogen bonds were observed with PHE (217), SER (475), VAL (145), and ARG(144). For STAT3, CA interacted with SER (514), GLN (511), ASP (334), and THR (515); THR (515) also contributed to ligand stabilization via hydrophobic interactions. Additionally, CA formed hydrogen bonds with ASN (103), PRO (80), MET (104), CYS (44), and PHE (43) of TGFB1.

### CA activates the SIRT1/FOXO1 pathway to induce autophagy and modulate lipid metabolism in the liver

**Fig. 5:**
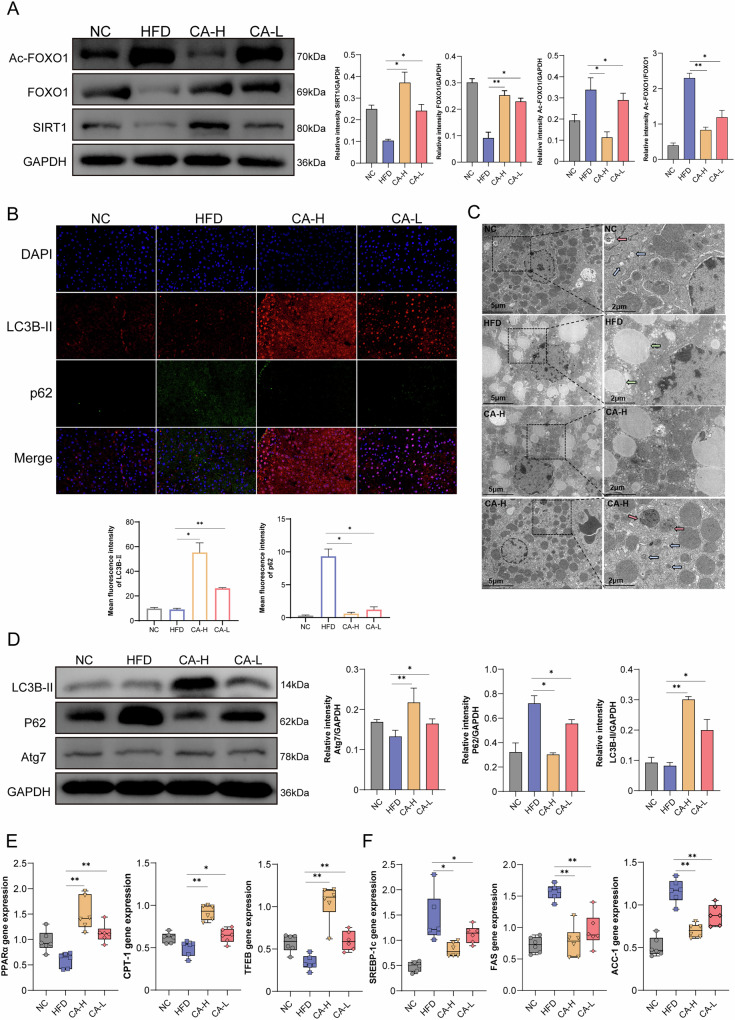
CA activates the SIRT1/FOXO1 pathway to induce autophagy and modulate lipid metabolism in the liver. **A** Protein expression and quantitative analysis of Atg7, p62, and LC3B-II in liver tissue (*n* = 3). **B** IF and quantitative analysis of LC3B and P62 in liver tissue. Scale bars: 20 μm (*n* = 3). **C** Representative TEM images of liver tissues from NC, model, CMA-treated mice, and CA-H groups are shown; CA-L images were omitted for clarity. Arrows denote the lipid drops (green arrow), autophagosomes (blue arrow), and autolysosomes (red arrow) structures. Scale bars: 5 and 2 μm. **D** Protein expression and quantitative analysis of SIRT1, FOXO1, and Ac-FOXO1 in liver tissue (*n* = 3). **E** The mRNA expression levels of fatty acid oxidation genes (PPARα, CPT-1, TFEB) in liver tissue (*n* = 6). **F** The mRNA expression levels of lipid synthesis genes (SREBP-1c, FAS, and ACC-1) in liver tissue (*n* = 6). Data are shown as the means ± SEMs. Compared with the HFD group, *P < 0.05, **P < 0.01.

To explore the potential association of the SIRT1/FOXO1 pathway in CA-mediated amelioration of MASLD, we quantified pathway-related protein expression through qRT-PCR and WB analysis. As illustrated in Fig. S2A, CA-treated mice demonstrated marked up-regulation of hepatic SIRT1 and FOXO1 mRNA expression levels. Consistent with transcriptional changes, WB analysis revealed substantial up-regulation of SIRT1 and FOXO1 proteins, along with down-regulation of Ac-FOXO1 protein, leading to a marked decrease in the Ac-FOXO1/FOXO1 ratio in hepatocytes of CA-treated mice (Fig. 5A). We next investigated whether autophagy mediates CA’s regulatory effects on lipid metabolism in HFD-induced MASLD mice. As illustrated in Fig. 5D, CA modulated key autophagy markers, demonstrating downregulation of p62 protein expression while upregulating LC3B-II and Atg7 protein levels compared to the model group. IF analysis further corroborated these findings, showing enhanced LC3B-II expression and reduced p62 levels following CA treatment (Fig. 5B). Furthermore, TEM analysis showed a significant accumulation of large cytosolic LDs (green arrow) in HFD-group hepatocytes. Conversely, CA-treated hepatocytes displayed small dense regions on the surface of large LDs, along with small LDs encapsulated within autophagosomes (blue arrow) or autolysosomes (red arrow) (Fig. 5C). These findings indicate that CA activates the SIRT1/FOXO1 pathway to induce autophagy.

Autophagy alleviates hepatic lipid accumulation by degrading lipid droplets and releasing FFAs into mitochondria for β-oxidation^27^. Interestingly, we demonstrated that CA administration increased the mRNA expression of markers of fatty acid oxidation (FAO), including peroxisome proliferator-activated receptor alpha (PPARα), carnitine palmitoyltransferase-1 (CPT1), peroxisomal acyl-coenzyme A oxidase 1 (ACOX1), and transcription factor EB (TFEB), whereas it downregulated the lipogenesis markers, such as peroxisome proliferator-activated receptor γ (PPARγ), FAS, and ACC-1 in the liver of MASLD mice (Fig. 5D,E). These findings indicate that CA potentially activates the SIRT1/FOXO1 pathway to induce autophagy and modulate lipid metabolism in the liver, thereby alleviating hepatic lipid accumulation.

### 3-MA/NAM treatment attenuates CA-mediated lipid clearance and antioxidant activity in vitro by inducing autophagy

**Fig. 6:**
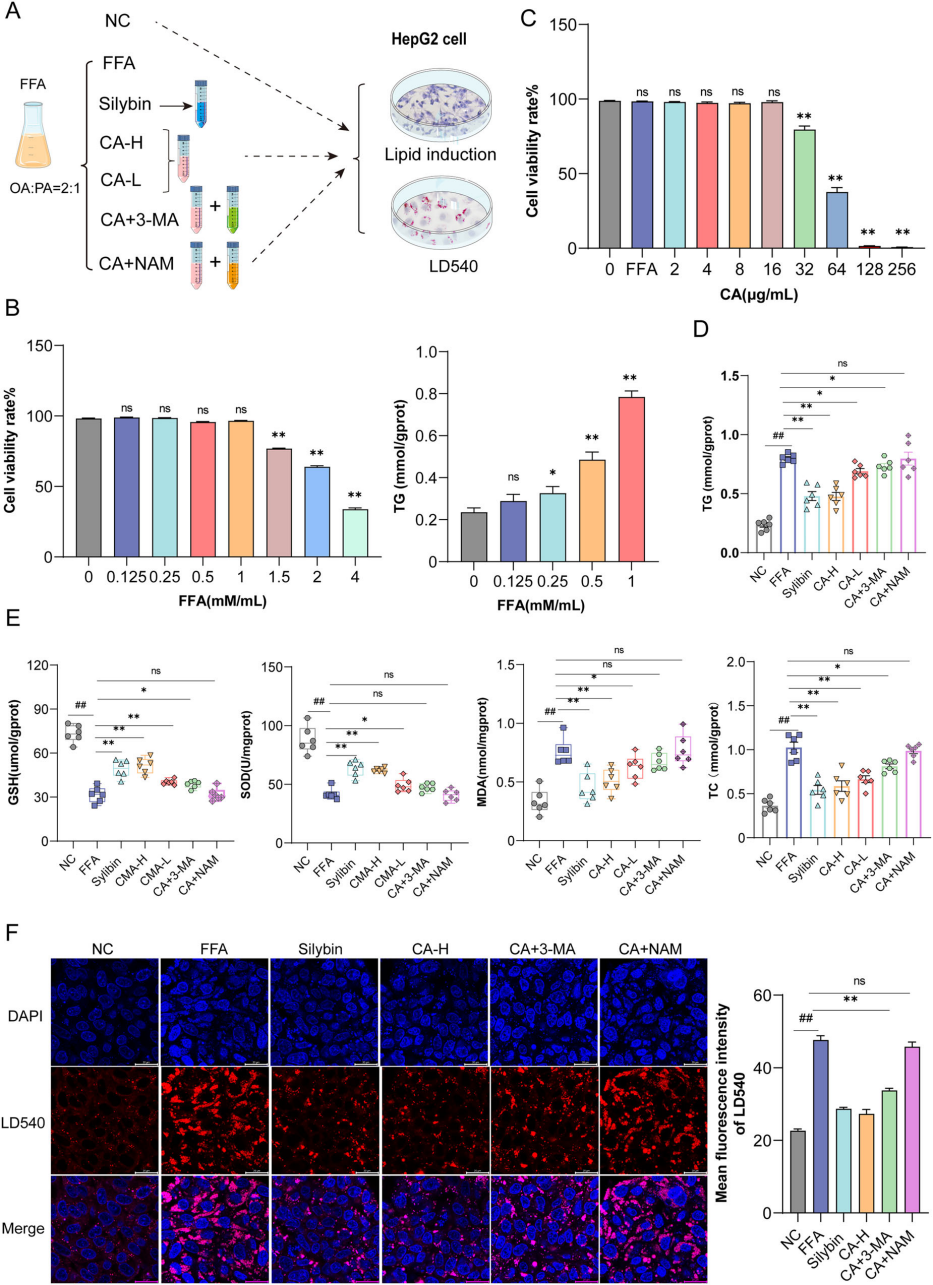
CA reduces FFA-induced lipid deposition in HepG2 cells. **A** Experimental outline. **B** Cell viability rate of HepG2 cells and intracellular TG levels in different concentrations of FFA. **C** Cell viability rate of HepG2 cells in different concentrations of CA with 1 mM FFA. **D** Intracellular TG and TC levels. **E** Intracellular GSH, SOD, and MDA levels. **F** LD540 fluorescence staining of intracellular lipid drops and quantified data are shown in the bottom panel. Scale bars: 20 μm. Data are shown as the means ± SEMs (*n* = 6). Compared with the NC group, ^#^*P* < 0.05, ^##^*P* < 0.01; compared with the FFA group, *P < 0.05, **P < 0.01.

To further investigate the underlying mechanisms of CA, we then investigated CA’s effects on FFA (PA:OA = 2:1)-induced lipid accumulation models in HepG2 cells (Fig. 6A). As shown in Fig. 6C, cell viability assays revealed that CA, at concentrations up to 16 μg/mL, did not exhibit cytotoxicity in the presence or absence of 1.0 mM FFA. A significantly elevated TG level was observed in cells induced by FFA at concentrations above 0.25 mM (Fig. 6B). Based on these results, we chose to use 1 mM FFA to induce cellular lipid accumulation that recapitulates features of MASLD.

To elucidate the regulatory role of the SIRT1/FOXO1 axis in CA-triggered autophagy, we co-treated cells with CA and the autophagy inhibitor 3-MA or the SIRT1 inhibitor NAM. CA concentration-dependently declined FFA-induced lipid accumulation (TG and TC) in HepG2 cells as determined by LD540 fluorescence staining. However, the lipid-lowering effect of CA was highly attenuated by 3-MA and completely abolished by NAM treatment (Fig. 6D,F). Additionally, CA mitigated FFA-induced oxidative stress, increasing GSH and SOD levels while reducing MDA, and these antioxidant effects were partially reversed by 3-MA and completely inhibited by NAM treatment (Fig. 6E).

**Fig. 7:**
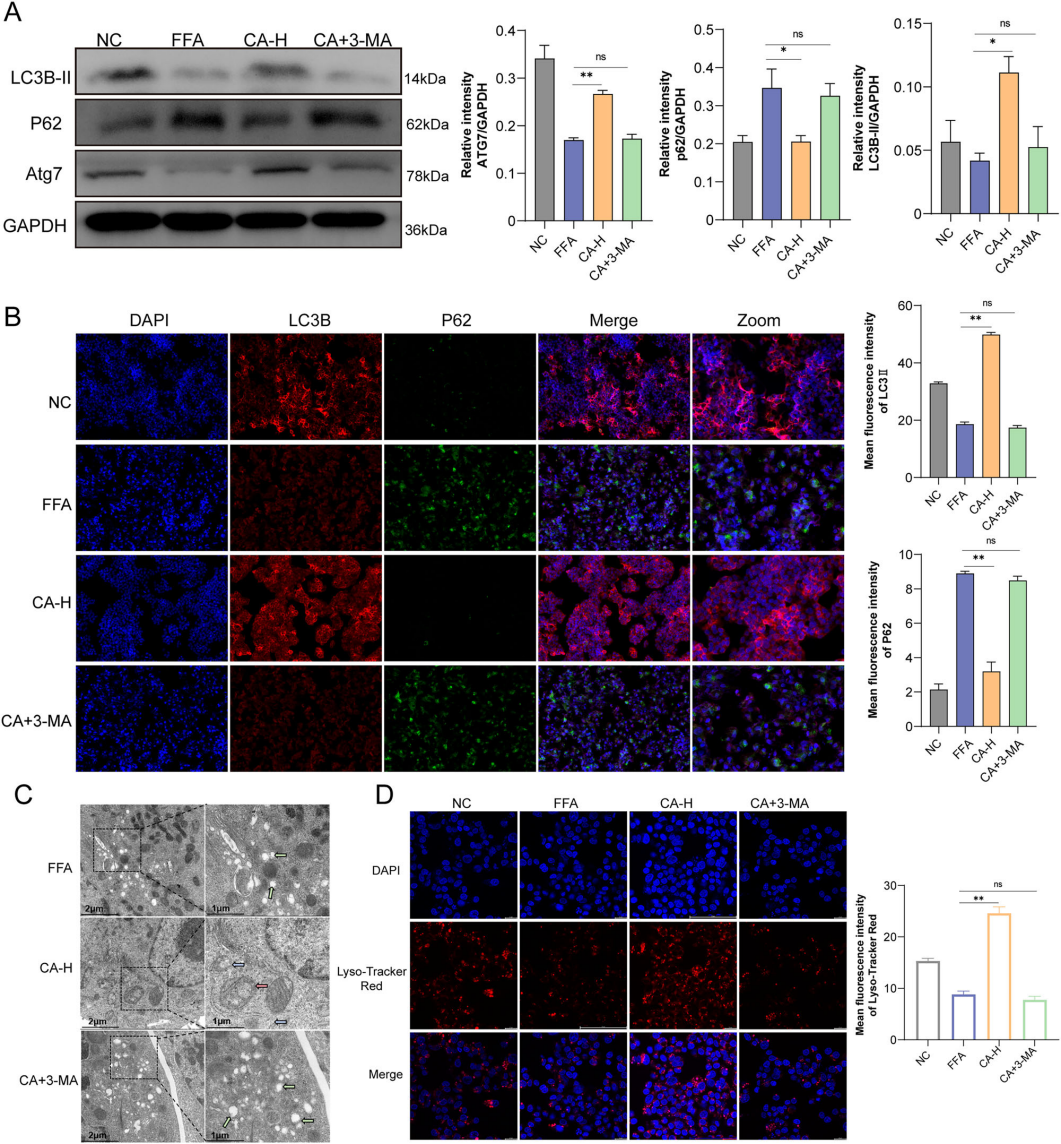
Treatment with 3-MA inhibits CA-induced autophagy in HepG2 cells. **A** Protein expression and quantitative analysis of Atg7, p62, and LC3B-II in HepG2 cells (*n* = 3). **B** IF and quantitative analysis of LC3B and p62 in FFA-exposed HepG2 cells. Scale bars: 20 μm (*n* = 3). **C** Representative TEM images of HepG2 cells from FFA, CA, and CA + 3-MA groups. Arrows denote the lipid drops(green arrow), autophagosomes (blue arrow), and autolysosomes (red arrow) structures. Scale bars: 2 and 1 μm. **D** Evaluation of the number of lysosomes using Lyso-Tracker Red fluorescent probes in HepG2 cells. Scale bars: 20 μm (*n* = 3). Data are shown as the means ± SEMs. Compared with the FFA group, *P < 0.05, **P < 0.01.

Additionally, as demonstrated in Fig. 7A and B, treatment with CA in the presence of FFA for 24 h significantly upregulated the expression of autophagy-related proteins (ATG7 and LC3B-II) while downregulating p62 levels in HepG2 cells. This CA-induced autophagic activation was further validated by IF staining of LC3B and p62. In contrast, co-treatment with CA and 3-MA resulted in a marked downregulation of ATG7 and LC3B-II levels, accompanied by an upregulation of p62, compared to CA treatment alone. The inhibitory effect of 3-MA on CA-mediated autophagy induction was further validated through IF staining of LC3B and p62. Meanwhile, autophagic flux was monitored using the lysosome red fluorescent probes (Lyso-Tracker Red). CA treatment markedly enhanced lysosomal biogenesis in FFA-exposed HepG2 cells. In contrast, 3-MA administration largely suppressed the fusion of autophagosomes with lysosomes, as evidenced by the diminished Lyso-Tracker Red signal (Fig. 7D). Ultrastructural examination through TEM revealed significant intracellular accumulation of large LDs (green arrow) in FFA-treated HepG2 cells. CA administration markedly attenuated the number of LDs while promoting autophagic flux, as indicated by increased autophagosome (blue arrow) and autolysosome (red arrow) formation in FFA-induced HepG2 cells (Fig. 7C). To further validate autophagic flux integrity, we employed the lysosomal inhibitor Bafilomycin A1 (Baf-A1). Similar to 3-MA, Baf-A1 co-treatment significantly attenuated, but did not fully abolish, CA-induced reductions in intracellular TG/TC and increases in GSH/SOD (Fig. S3A). These results inversely confirm that the beneficial effects of CA on MASLD are linked to enhanced autophagy.

### CA activates SIRT1/FOXO1 signaling to stimulate autophagy-dependent FAO and inhibit lipogenesis

NAM, a niacin-derived amide compound, acts as a potent inhibitor of SIRT1 deacetylation activity, resulting in elevated acetylation levels of FOXO1 and subsequent suppression of its transcriptional activity^28^. This reduction in FOXO1 transcriptional activity downregulates key autophagy-related genes (such as LC3 and ATG7), ultimately impairing autophagosome formation and disrupting autophagic flux. Next, we assessed the expression of pathway-related proteins using WB, RT-qPCR, and IF staining. As illustrated in Fig. S2B, CA up-regulated the SIRT1 and FOXO1 mRNA expression levels in FFA-induced HepG2 cells.

**Fig. 8:**
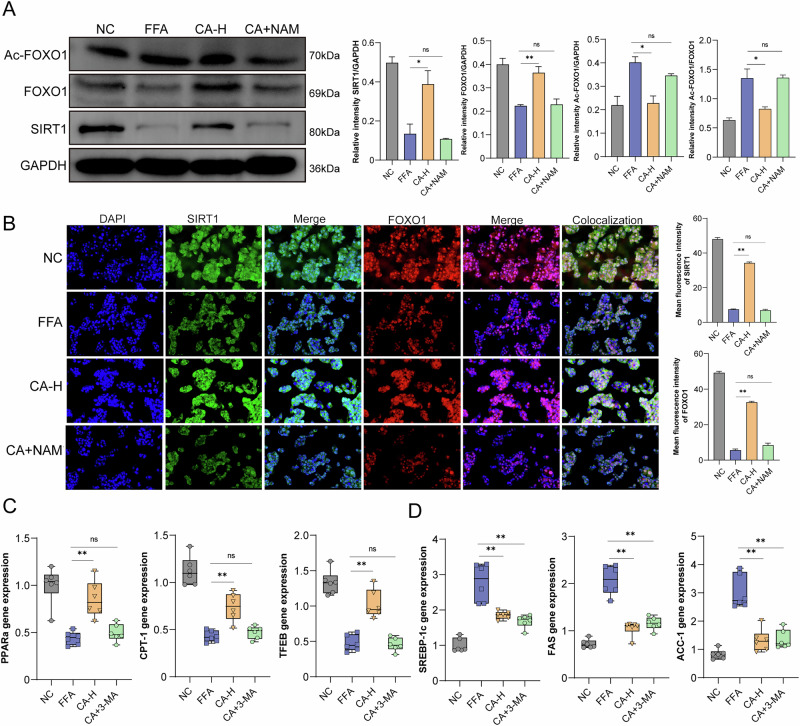
CA activates the SIRT1/FOXO1 to modulate lipid metabolism in HepG2 cells. **A** Protein expression and quantitative analysis of SIRT1, FOXO1, and Ac-FOXO1 in HepG2 cells (*n* = 3). **B** IF and quantitative analysis of SIRT1 and FOXO1 in HepG2 cells. Scale bars: 20 μm (*n* = 3). **C** The mRNA expression levels of fatty acid oxidation genes (PPARα, CPT-1, and TFEB) in HepG2 cells (*n* = 6). **D** The mRNA expression levels of lipid synthesis genes (SREBP-1c, FAS, and ACC-1) in HepG2 cells (*n* = 6). Data are shown as the means ± SEMs. Compared with the FFA group, *P < 0.05, **P < 0.01.

Consistent with transcriptional changes, WB analysis revealed substantial up-regulation of SIRT1 and FOXO1 proteins, along with down-regulation of AC-FOXO1 protein, resulting in a marked reduction of the Ac-FOXO1/FOXO1 ratio in CA-treated HepG2 cells compared to the model group (Fig. 8A). Under basal conditions, FOXO1 predominantly localizes to the nucleus, modulating gene transcription. SIRT1-mediated deacetylation enhances the transcriptional activity and nuclear retention of FOXO1, thereby upregulating the expression of autophagy-related genes and promoting autophagic flux^29^. Consistently, WB analysis of subcellular fractions revealed that CA treatment promoted nuclear accumulation of FOXO1, which was effectively abolished by NAM (Fig. S4). IF staining revealed that CA treatment markedly upregulated SIRT1 and FOXO1 expression and enhanced their cytoplasmic co-localization in HepG2 cells (Fig. 8B). However, the administration of the SIRT1 inhibitor NAM attenuated the stimulatory effect of CA on the SIRT1/FOXO1 pathway.

Consistent with our early findings, CA upregulated mRNA expression of key genes regulating FAO (PPARα, CPT-1, and TFEB) and downregulated those involved in lipid synthesis (SREBP-1C, FAS, and ACC-1). However, 3-MA treatment abolished the CA-induced promotion of FAO while leaving the inhibitory effect of CA on lipid synthesis unaffected (Fig. 8C,D). Similarly, co-treatment with BafA1 also suppressed the CA-induced upregulation of the fatty acid oxidation genes PPARα and CPT-1 (Fig. S3B).

**Fig. 9:**
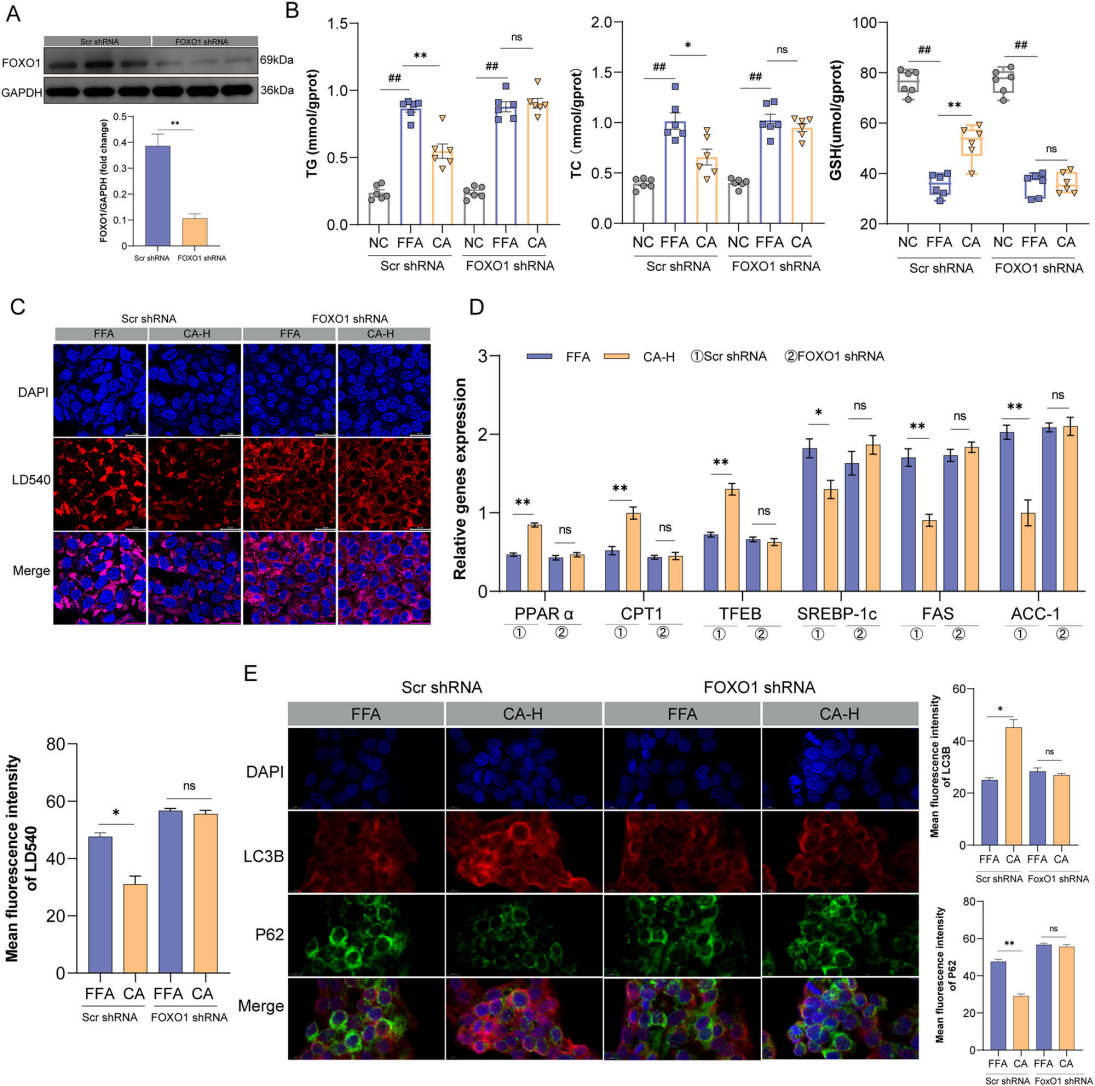
Effects of FOXO1 knockdown in HepG2 cells. **A** FOXO1 knockdown in HepG2 cells was achieved by transfecting with shRNA against FOXO1. The protein level of FOXO1 was determined by immunoblotting. Scr scrambled shRNA. **B** Intracellular TG, TC, and GSH levels. **C** LD540 fluorescence staining of intracellular lipid drops and quantified data are shown in the bottom panel. Scale bars: 20 μm. **D** Relative gene expression in HepG2 cells with FOXO1 gene knockdown. **E** IF and quantitative analysis of LC3B and p62 in FFA-exposed HepG2 cells. Scale bars: 10 μm (*n* = 3). Data are shown as the means ± SEMs. Compared with the FFA group, *P < 0.05, **P < 0.01.

To further validate the necessity of FOXO1, we generated FOXO1-knockdown cells (Fig. 9A). Results showed that FOXO1 knockdown markedly blunted CA’s lipid-lowering and antioxidant effects, as evidenced by increased intracellular lipid droplet content, elevated TG and TC levels, and reduced GSH activity (Fig. 9B,C). Moreover, CA lost its ability to regulate LC3B and p62 protein expression, failed to effectively induce autophagy, and exhibited significantly impaired modulation of downstream lipid metabolism genes (Fig. 9D,E). These findings indicate that CA attenuates hepatic lipid synthesis while promoting autophagy-dependent fatty acid oxidation through activation of the SIRT1/FOXO1 signaling axis.

### CA altered the gut microbiota composition

**Fig. 10:**
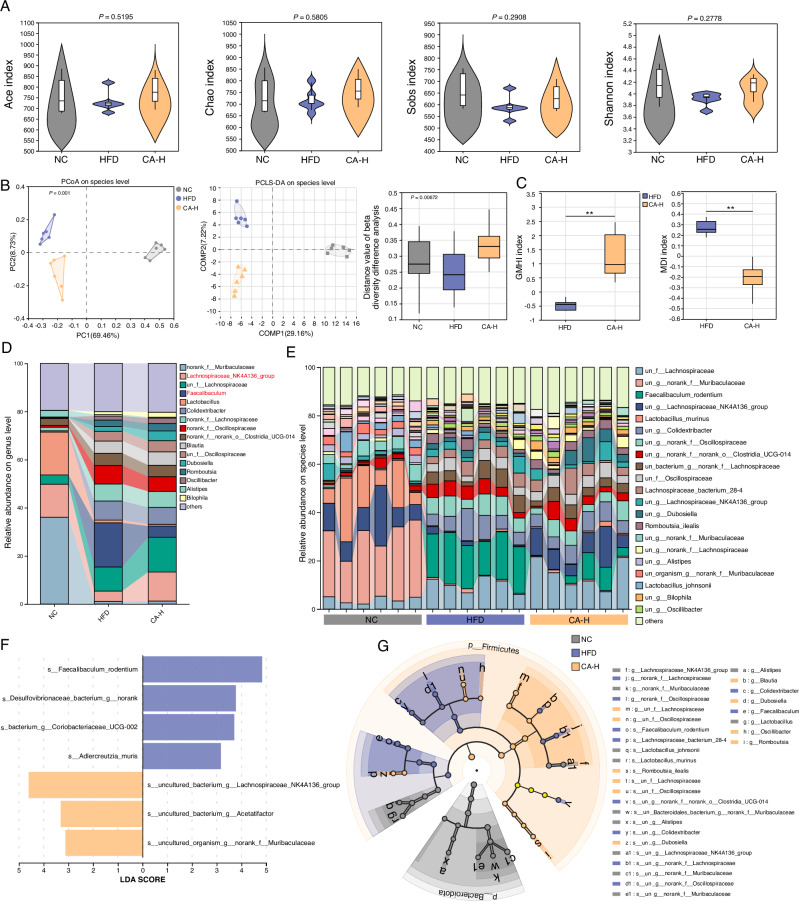
CA alters the gut microbiota in HFD-fed mice. **A** Ace index, Chao index, Sobs index, and Shannon index of α-diversity. **B** PCoA and PCLS-Da on the species level. Distance value of beta diversity difference analysis. **C** GMHI and MDI of fecal microbiota. **D** Community barplot analysis of fecal microbiota composition in HFD and CA-treated mice at genus levels. **E** Community barplot analysis of fecal microbiota composition in each HFD and CA-treated mouse at the species level. **F** LDA score representing the taxonomic data with a significant difference between the two groups. Only LDA scores >3 are shown. Yellow indicates enriched taxa in the CA group. Blue indicates enriched taxa in the HFD group. **G** Analysis of the relative abundance of taxa among different groups using a LEfSe cladogram. Circle sizes in the cladogram plot are proportional to bacterial abundance. The circles represent, going from the outer to the inner circle: species, genus, class, order, family, and phylum. LDA ≥ 3.0 and *p* ≤ 0.05 (Wilcoxon rank-sum test) was regarded as significant. Data are shown as the means ± SEMs (*n* = 6). Compared with the HFD group, *P < 0.05, **P < 0.01.

Gut microbiota has been demonstrated to be strongly intertwined with the development of MASLD, MASH, and cirrhosis. To determine whether CA modulates the gut microbiota in the alleviation of MASLD, the composition of the intestinal microbiota was analyzed via 16S rRNA sequencing. There was no significant difference between CA-treated and HFD mice in α-diversity, as indicated by the Ace, Chao, Shannon, and Sobs indices (Fig. 10A), whereas PCoA, PCLS-DA and beta diversity analysis showed significantly different clustering of gut microbiota in mice of two groups (Fig. 10B). Furthermore, we found that CA group has a much higher gut microbiota health index (GMHI) and a lower microbial dysbiosis index (MDI), a set of indicators used to assess the ecological health of microorganisms based on species-level taxonomic characteristics (Fig. 10C).

**Fig. 11:**
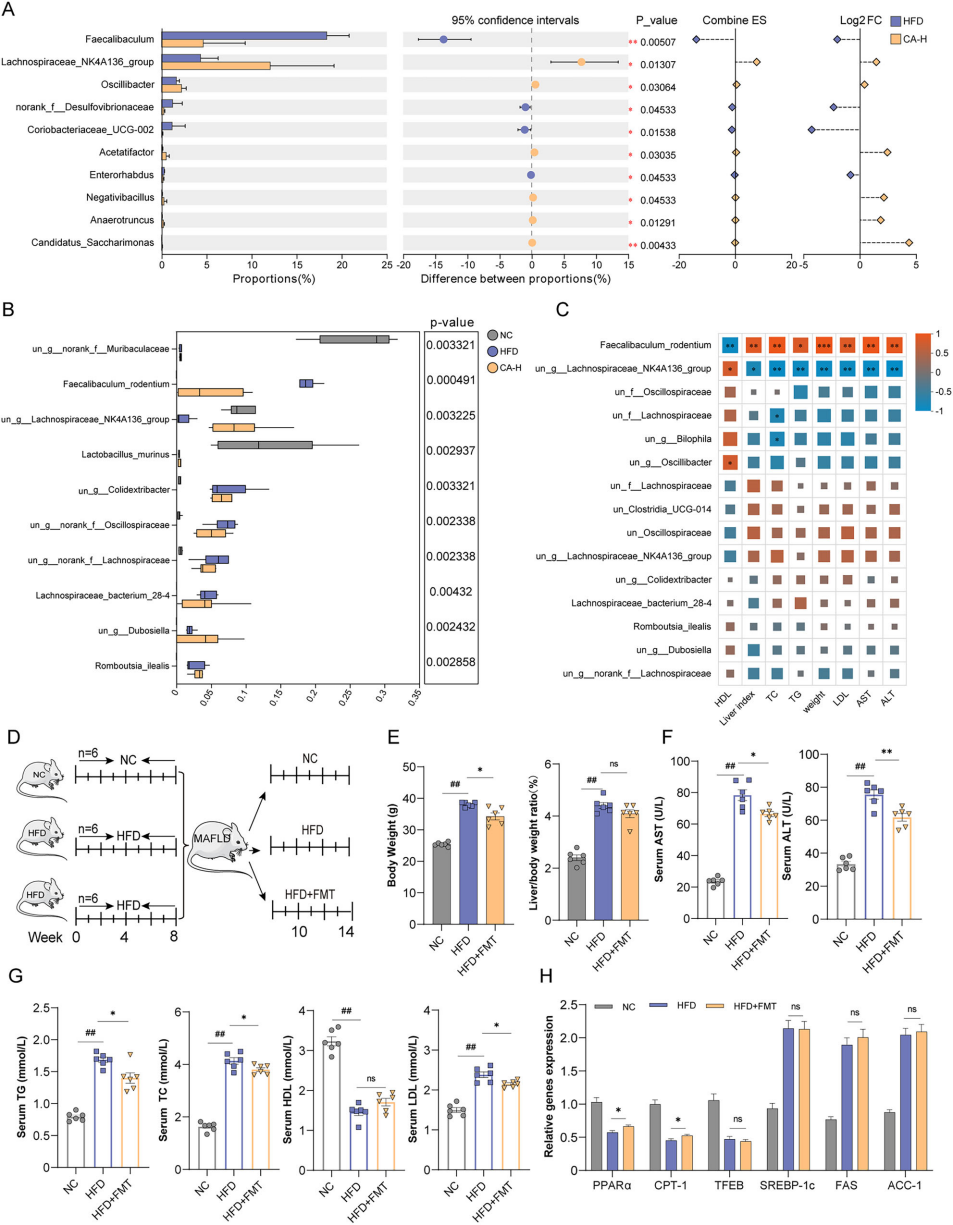
Altered gut microbiota in CA-modified mice exhibits beneficial effects on MASLD. **A** Wilcoxon rank-sum test bar plot on the genus level in CA-H and HFD group mice; CA-H‌ stands for the ‌high-dose group of CA in mice‌. **B** Kruskal–Wallis *H* test bar plot on the spice level in CA-H and HFD group mice. **C** Correlation analysis of body weight, liver index, and serum TG, TC, HDL, LDL, AST, and ALT levels with the abundance of *Faecalibaculum_rodentium* and *un_g__ Lachnospiraceae_NK4A136_group* in CA-H and HFD group mice. **D** Schematic of the animal experiment. C57BL/6J mice were randomly divided into NC, HFD, and FMT groups after 8 weeks on a high-fat diet. **E** Liver weight and the ratio of liver weight to body weight. **F** Serum AST and ALT. **G** Serum TG, TC, HDL, and LDL. **H** Relative gene expression in the livers (*n* = 6). Data are shown as the means ± SEMs (*n* = 6). Compared with the HFD group, *P < 0.05, **P < 0.01.

In addition, community barplot analysis and Linear discriminant analysis (LDA) effect size (LEfSe) analysis revealed that the genera *Faecalibaculum* and *Coriobacteriaceae UCG 002 group*, and the species *Faecalibaculum rodentium* and *Desulfovibrionaceae* bacterium were enriched in the HFD group, whereas the families *Lachnospiraceae*, and the genera *Acetatifactor* and *Lachnospiraceae_bacterium_28-4* were increased in the gut of CA-treated HFD mice. Statistical analysis showed that CA intervention significantly ameliorated the high-fat diet-induced reduction in the abundance of *Lachnospiraceae NK4A136 group*, while markedly attenuating the elevation of *Faecalibaculum* genus abundance (Fig. 10D–G). Intriguingly, these two genera exhibited substantial effect sizes, which may be identified as the major contributors to the induced changes in the phenotypic factors of MASLD by CA (Fig. 11A and B). Correlation analyses revealed that the abundance of *Lachnospiraceae NK4A136 group* was inversely correlated with murine body weight, TC, TG, AST, and related metabolic markers, whereas Faecalibaculum displayed an opposing trend (Fig. 11C). Notably, *Lachnospiraceae NK4A136 group* is typically associated with short-chain fatty acid (SCFA) production, and its depletion may exacerbate metabolic dysregulation through diminished microbial-derived SCFA bioavailability. Collectively, CA exerts a protective effect on the liver by altering the gut microbiome, and the insightful correlations provide a strong rationale for further investigation into the potential health benefits associated with these enriched bacteria.

To further confirm whether the gut microbiota of mice following CA modulation exerts beneficial effects against MASLD, we performed FMT experiments and measured short-chain fatty acids in feces (Fig. 11D). As shown in Fig. 11E–G, FMT intervention reduced body weight, hepatic transaminases, and serum lipid levels in the model mice. RT-qPCR analysis revealed that FMT upregulated hepatic expression of PPARα and CPT-1, thereby enhancing hepatic FAO (Fig. 11F). Furthermore, CA intervention significantly increased butyrate levels, which showed a positive correlation with the abundance of the *Lachnospiraceae NK4A136 group* (Fig. S5). Collectively, these findings indicate that CA-mediated modulation of the gut microbiota partially contributes to the improvement of MASLD.

## Discussion

MASLD is closely linked to metabolic syndrome, central obesity, type 2 diabetes mellitus (T2DM), and dyslipidemia. Autophagy, an evolutionarily conserved lysosomal degradation pathway, eliminates dysfunctional organelles, protein aggregates, and excess macromolecules, thereby maintaining cellular homeostasis, mitigating metabolic stress, and ensuring energy balance^30^. In the liver, autophagy is crucial for maintaining lipid homeostasis and limiting pathological lipid accumulation, making its pharmacological activation a potential therapeutic strategy for metabolic liver disorders^31,32^. This study aimed to explore the protective mechanisms of CA in MASLD. Preliminary results indicate that CA alleviates MASLD phenotypes, including reduced body weight, hepatic steatosis, and serum lipid levels, while enhancing antioxidant capacity and suppressing inflammation. Through bioinformatics, network pharmacology, and experimental validation, we demonstrate that CA mitigates hepatic steatosis by activating SIRT1, promoting FOXO1 deacetylation, inhibiting lipid synthesis, and inducing autophagy to promote FAO, highlighting its role as a potent autophagy inducer.

MASLD is pathologically characterized by excessive hepatic lipid accumulation, rendering the modulation of hepatic lipid metabolism a critical therapeutic target for this condition^33^. Extensive experimental evidence has demonstrated that HFD-induced MASLD models consistently exhibit marked elevations in both serum and intrahepatic lipid profiles^34^. CA, the main active ingredient in the bark of cinnamon, belongs to aromatic aldehydes, which have been widely used in both food and drug formulations in Asia, with anti-inflammatory, antioxidant, and lipid-lowering^11,35^ features that have been further confirmed by our study. Insulin resistance is a key driver in the pathogenesis of MASLD and its progression from steatosis to steatohepatitis^36^. Notably, our study revealed that CA effectively ameliorates insulin resistance and hyperglycemia in MASLD mice. Considering CA did not alter food intake or induce observable side effects, we believe that CA may serve as an ideal candidate drug to treat MASLD.

The SIRT1/FOXO1 pathway regulates autophagy activity through deacetylation modification, targets lipid droplet degradation, and inhibits lipid synthesis, which is a key mechanism to ameliorate lipid metabolism disorders^37^. Notably, CA enhanced hepatic autophagy by activating the SIRT1/FOXO1 axis, as demonstrated by upregulated expression of SIRT1, FOXO1, LC3B-II, and ATG7 (key autophagy regulators), along with autophagic degradation of p62 and Ac-FOXO1 in vivo. TEM analysis revealed that CA intervention preserved the fundamental cellular structure, augmented the quantity and dimensions of mitochondria, and enhanced the number of autophagosomes. Further investigations revealed that CA treatment significantly upregulated the expression of key fatty acid oxidation genes (PPARα, CPT-1, and TFEB) while downregulating lipogenic genes (SREBP-1c, FAS, and ACC), ultimately attenuating hepatic lipid accumulation. These findings demonstrate that CA facilitates cellular metabolic reprogramming through SIRT1/FOXO1 pathway activation, which orchestrates autophagy induction and lipid metabolism regulation, thereby maintaining hepatic energy homeostasis.

Next, we employed 3-methyladenine (3-MA), a pharmacological inhibitor of autophagosome formation, to further investigate the mechanistic role of CA-induced autophagy in lipid metabolism^38^. ‌FOXO1 induces the expression of LC3-II protein, which facilitates the formation of lipophagosomes that encapsulate lipid droplets via autophagosome-mediated processes. Following fusion with lysosomes, lipophagosomes release lipases to hydrolyze triglycerides into FFAs, providing energy for mitochondrial β-oxidation and reducing lipid accumulation in hepatocytes^39^. Utilizing the LD540 fluorescent probe, we observed that CA significantly diminished FFA-induced red-labeled lipid droplets in HepG2 cells. However, 3-MA treatment effectively abrogated both the hypolipidemic effects of CA and its attenuation of lipid peroxidation in FFA-treated HepG2 cells. Ultrastructural analysis by TEM further demonstrated that CA-induced autophagy specifically facilitated the lysosomal degradation of lipid droplets in these cells. Furthermore, 3-MA inhibited CA-induced autophagy as indicated by decreased LC3B-II and ATG7 protein expression, increased P62 protein expression, and reduced number of intracellular lysosomes. Emerging evidence has revealed that autophagy-mediated lipid catabolism facilitates FAO and ketogenesis, thereby maintaining hepatic energy homeostasis. Conversely, disruption of selective autophagy pathways markedly‌ impairs both FAO capacity and ketone body synthesis, exacerbating MASLD progression^40,41^. 3-MA treatment mitigated CA-induced upregulation of PPARα, CPT-1, and TFEB gene expression, indicating that CA enhances FAO in an autophagy-dependent manner.

SIRT1 serves as a key regulator of glucose/lipid homeostasis, inflammatory signaling, cellular senescence, and apoptosis, thereby mediating hepatoprotective effects against metabolic-associated liver disorders. As a downstream target of SIRT1, FOXO1 is deacetylated and activated, thereby inhibiting inflammation and oxidative stress^42^. To further verify the essential role of CA in hepatic lipid metabolism associated with activation of the SIRT1/FOXO1 pathway, NAM and shRNA-FOXO1 were used to inhibit SIRT1 and FOXO1 activity. As guessed, NAM intervention abolished the ameliorating effect of CA on FFA-induced lipid deposition and oxidative stress in HepG2 cells. SIRT1 is mainly expressed in the nucleus and cytoplasm, while FOXO1 is mainly located in the nucleus. This finding is consistent with previous studies, SIRT1 induces autophagy and regulates lipid metabolism by activating FOXO1 to deacetylate and translocate it into the nucleus^43,44^. Genetic evidence revealed that FOXO1 knockout mice showed significant hepatic steatosis and liver injury^45^. IF showed that NAM reduced CA-induced expression of SIRT1 and FOXO1 and their co-localization in the nucleus. In addition, 3-MA had no effect on the inhibitory effect of CA on lipid synthesis. These findings indicate that CA-mediated activation of the SIRT1/FOXO1 pathway directly inhibits hepatic lipid synthesis.

Growing evidence highlights gut microbiota dysbiosis as a key modulator of MASLD pathogenesis and disease progression, mediated through inflammatory response activation and metabolic dysregulation^46,47^. The GMHI and MDI are employed to evaluate the integrity and compositional balance of the intestinal microbial ecosystem. Notably, MASLD patients typically demonstrate elevated GMHI levels and reduced MDI values, indicative of profound gut microbiota dysbiosis^48^. In our study, CA intervention markedly elevated the GMHI while significantly reducing the MDI, effectively ameliorating gut microbiota dysbiosis. Furthermore, CA intervention decreased the abundance of *Faecalibaculum*, which exhibited a strong and consistent positive correlation with the release of pro-inflammatory factors^49^. Correlation analysis further revealed that MASLD-promoting parameters were significantly and positively associated with *Faecalibaculum*. In contrast, the *Lachnospiraceae_NK4A136_group*, a potentially beneficial bacterium, produces short-chain fatty acids through dietary polysaccharide fermentation, which were inversely correlated with chronic inflammation and metabolic disorders^50^. Similarly, treatment immensely enriched the abundance of the *Lachnospiraceae_NK4A136_ group*, showing a negative correlation with MASLD-promoting indicators but a positive correlation with serum HDL levels. Subsequently, FMT experiments further confirmed that CA can partially ameliorate MASLD by modulating the gut microbiota in mice. Furthermore, the elevation of fecal butyrate levels also illustrates the functional role of the enriched *Lachnospiraceae_NK4A136_group* at the metabolite level. Future studies employing germ-free or antibiotic-based models would be valuable to fully establish causality.

**Fig. 12:**
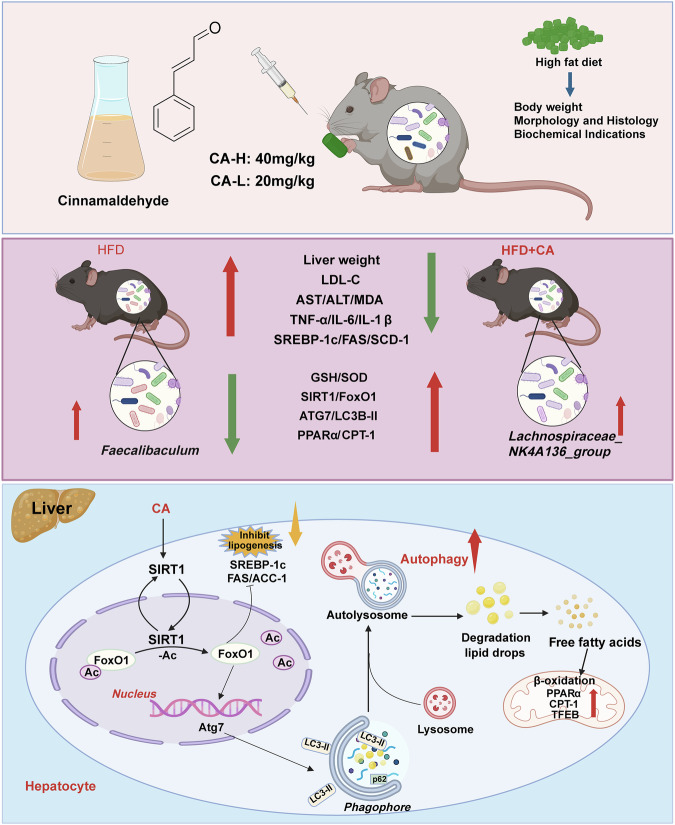
Potential mechanism of action of CA in MASLD treatment. This diagram was constructed using BioRender (https://www.biorender.com/).

In conclusion, CA ameliorates hepatic steatosis by activating the SIRT1/FOXO1 signaling pathway to suppress lipogenesis and enhance autophagic flux to promote FAO (Fig. 12). Moreover, pharmacological inhibition of the autophagy-lysosomal pathway impaired the lipid-lowering activity of CA, which mainly involved autophagy and was partially related to the activation of FAO in vivo. Meanwhile, CA restored gut microbiota homeostasis in MASLD mice. This study advanced our understanding of the molecular basis of using CA for treating MASLD and provided substantial pharmacological evidence supporting CA as a potential therapeutic candidate for MASLD attenuation. Of course, it is recognized that pharmacokinetic, bioavailability, and hepatic concentration data are crucial for a comprehensive understanding of CA’s therapeutic potential. Relevant experiments conducted in future studies may address concerns regarding dosing safety and data completeness, thereby providing a more robust foundation for understanding CA’s therapeutic potential and guiding future clinical applications. Additionally, the study’s exclusive use of male mice may limit the generalizability of our findings to females, who exhibit distinct metabolic and inflammatory profiles in MASLD. However, this design choice was intentional to ensure robustness of the primary endpoint.

## Material

### Reagents and materials

60% high-fat diet was purchased from Jiangsu Xietong Pharmaceutical Bio-engineering Co., Ltd. (Nanjing, China). CA was purchased from Aladdin Reagent (CAS: 104-55-2). The sodium palmitate and oleic acid were procured from Shanghai McLean Biochemical Technology Co., Ltd. Triacylglycerol (TG), cholesterol (TC), aspartate transaminase (AST), alanine transaminase (ALT), high-density lipoprotein (HDL), low-density lipoprotein (LDL), free fatty acid (FFA) glutathione (GSH), superoxide dismutase (SOD), malondialdehyde (MDA), thiobarbituric acid reactive substances (TBARs), tumor necrosis factor-α (TNF-α), interleukin 6 (IL-6), and interleukin-1 beta (IL-1β) ELISA kits were purchased from Nanjing Built Bioengineering Institute (Nanjing, China). The total RNA extraction reagents (FastPure Cell/Tissue Total RNA Isolation Kit V2) were procured from Nanjing Vazyme Biotechnology Co., Ltd. The cell lipid drops staining kit, cDNA kit SweScript All-in-One RT SuperMix for qPCR (One-Step gDNA Remover), and reverse transcription kit 2×Universal Blue SYBR Green qPCR Master Mix were purchased from Servicebio (Wuhan, China). Cell counting kit-8 (CCK-8) was purchased from Solarbio (Beijing, China). First antibodies SIRT1 (ab189494) and FOXO1 (ab179450) were purchased from Abcam, LC3Ⅱ (#12741), Atg7(#8558) and p62 (#5114) were obtained from CST, Ac-FOXO1 was purchased from Affinity (#AF2305). GADPH (10,494–1-AP) and secondary antibodies for Western blot (WB) were obtained from Servicebio (Wuhan, China).

### Animal experiments

All animal experimental procedures complied with the regulations of the People’s Republic of China on the management of experimental animals and were approved by the Animal Experimentation Ethics Committee of Henan University of Chinese Medicine (Zhengzhou, China) (Laboratory Animal Use License No. SYXK (Henan) 2021-0015) (Animal Ethics Approval Number IACUC-202401011). Male C57BL/6J mice (7-week-old, SPF-grade) were housed under controlled conditions (temperature: 22 ± 1 °C, humidity: 50 ± 10%, 12 h light/dark cycle) with ad libitum access to food/water. After acclimatization, mice were randomized into: (1) normal control (NC, *n* = 8, standard diet); (2) high-fat diet (HFD, *n* = 8); (3) silybin (50 mg/kg, *n* = 8); (4) CA-high (40 mg/kg, *n* = 8); and (5) CA-low (20 mg/kg, *n* = 8). CA groups received daily oral suspensions (0.5% CMC-Na vehicle) for 10 weeks, while NC/HFD groups received vehicle only. ‌Following overnight fasting, blood was obtained by removing the eyeball prior to sacrifice. Liver tissue and epididymal fat collection were immediately performed. Fecal samples were stored at –80 °C. Mice were euthanized by carbon dioxide inhalation. The animals were placed in a chamber and exposed to a flow of 100% CO₂ at a rate of 10–30% of the chamber volume per minute. Death was confirmed by the absence of respiratory movement, heartbeat, and corneal reflex. All procedures were performed by trained personnel to ensure humane treatment and minimize distress.

### Glucose tolerance testing(OGTT)

Following a 6-h fasting, mice received an intraperitoneal injection of glucose (2 g/kg body weight) at 9:00 a.m. Tail vein blood samples were collected at baseline (0 min) and at 15, 30, 60, and 120 min post-challenge to measure blood glucose levels using a FreeStyle Lite glucose monitoring system.

### Insulin tolerance test (ITT)

Following a 6-h fasting, mice received an intraperitoneal injection of insulin (1.0 U/kg body weight) at 9:00 a.m. Blood glucose concentrations were quantified via serial tail-vein sampling at baseline (0 min) and at 15, 30, 60, 90, and 120 min post-administration using a calibrated portable glucometer.

### Serum biomarkers

Biochemical parameters (AST and ALT) and lipid profiles (TC, TG, HDL, and LDL), along with inflammatory cytokines (IL-6, TNF-α, and IL-1β) in mouse serum, were analyzed using standardized ELISA kits based on the manufacturer’s protocols.

### Hepatic biomarkers

Total hepatic lipid extraction was performed using a tert-butyl alcohol/methanol/Triton X-100 (3:1:1, v/v/v) solvent system from 10% (w/v) liver tissue homogenates. Hepatic oxidative stress was evaluated by measuring GSH, SOD, and MDA levels. 10% liver homogenate was mixed with 0.1 ml chloroform and then vortexed and centrifuged. Hepatic TG, TC, FFA, GSH, MDA, SOD, and TBARs contents were quantified using assay kits following the manufacturer’s protocols.

### Morphology and histology

After 24-h fixation in 4% PFA, liver and adipose tissues were paraffin-embedded, sectioned (5 μm), and H&E-stained for histopathological examination. Masson’s trichrome staining was performed on adjacent paraffin sections to evaluate collagen deposition/fibrosis. NAFLD activity score (NAS) was assessed on H&E-stained liver sections according to standard criteria (steatosis, lobular inflammation, and hepatocellular ballooning). For Oil Red O staining (ORO), the fixed liver samples were embedded in optimum cutting temperature compound. Sections of 10 µm were stained with 0.5% Oil Red O solution to assess hepatic lipid accumulation. Whole-tissue slide scans at ×40 magnification were performed under a microscope slide scanner (Pannoramic MIDI, 3DHISTECH). Relative ×20 and ×80 images were analyzed with image-analyzing software CaseViewer_2.3. The staining of LC3Ⅱ and P62 in liver tissue for IF analysis was performed following a standard protocol.

### RNA-seq data analysis

RNA sequencing data were processed following established protocols. Initial quality control was performed using Trim Galore (v0.4.4) for read trimming. Differential gene expression was analyzed with DESeq2 (v1.24.0), followed by functional enrichment analysis using clusterProfiler (v3.12.0) for Gene Ontology annotation. The RNA-seq analysis was performed at the online platform Majorbio Cloud Platform (www.majorbio.com).

### Network pharmacology and molecular docking analysis

CA-related targets were predicted using TCMSP, PharmMapper, and SwissTarget Prediction databases. The resulting targets were then filtered through the Uniprot database to retain only reviewed human proteins (*Homo sapiens*). MASLD-related targets (score > 5) were extracted from GeneCards, DisGeNET, and OMIM databases. Common targets between CA and MASLD were identified via Venny 2.1.0. A protein–protein interaction (PPI) network was constructed using STRING and visualized in Cytoscape. Core targets were subjected to Gene Ontology (GO) and Kyoto Encyclopedia of Genes and Genomes (KEGG) pathway enrichment analysis using Metascape.

The crystallographic structures of AKT1, TGFB1, SIRT1, and STAT3 were obtained from RCSB PDB (https://www.rcsb.org/) for molecular docking. CB-Dock2 was employed for docking simulations^51^. The CAM ligand structure was generated using Chem3D and energy-minimized with the MMFF94 force field. The optimal docking pose was selected based on the lowest binding affinity score and visualized using PyMol 3.1.3.

### Transmission electron microscope (TEM)

An appropriate amount of fresh liver tissue, about 1 mm^3^, was taken and fixed in an electron microscope fixative solution. The tissues were rinsed 3 times with 0.1 mmol/L phosphate buffer (pH 7.4) for 15 min each time. Subsequently, post-fixation, dehydration at room temperature, osmotic embedding, polymerization, semi-thin section (1.5 μm), toluidine blue staining, localization under a light microscope, and ultrathin section (60–80 nm) were performed. The specimens were stained with 2% uranyl acetate saturated alcohol solution in the dark, washed, and 2.6% lead citrate solution in the dark. Ultrastructural analysis of hepatocytes was performed by TEM to evaluate autophagosome and autolysosome formation.

### Cell viability, lipids content, and oxidative stress detection

The HepG2 cells (American Type Culture Collection) were maintained in Dulbecco’s modified Eagle medium (Servicebio, China) supplemented with 15% fetal bovine serum (LONSERA, Uruguay) and 1% penicillin/streptomycin (Servicebio, China), under standard culture conditions (37 °C, 5% CO₂, humidified atmosphere). The cell viability was determined using a CCK-8 assay per the manufacturer’s protocol. 5 × 10³ cells/well were plated in 96-well flat-bottom plates. Following 24 h treatment with CA (2–256 μg/mL in 2-fold increments), 10 μl of CCK-8 solution was aliquoted into each well, and the absorbance density was measured at 450 nm on the spectrophotometer.

To mimic hepatic steatosis in vivo, HepG2 cells were maintained in a medium containing 1.0 mmol/l FFA (oleic acid, oleic acid to palmitic acid ratio 2:1) for 24 h, and treated with high or low doses of CA and SIRT1 inhibitor NAM or autophagy inhibitor 3-MA. The contents TG, TC, GSH, SOD, and MDA were quantified using the commercial kits. Meanwhile, intracellular lipid droplet accumulation was assessed by the LD540 fluorescent probe.

### Immunofluorescence and fluorescence microscopy

The HepG2 cells underwent sequential fixation with 4% paraformaldehyde (15 min), permeabilization using 0.2% Triton X-100 (10 min), and blocking with 5% BSA/PBS (1 h, RT) to minimize nonspecific binding. Primary antibodies against SIRT1, FOXO1, LC3Ⅱ, and P62 were applied overnight (4 °C). After PBS washes, species-matched secondary antibodies (FITC-conjugated goat Anti-Rabbit IgG; Cy3-conjugated goat Anti-Mouse IgG) were incubated (1 h, RT). Nuclei were visualized with 4’,6-diamidino-2- phenylindole (DAPI) for 5 min. Confocal fluorescence imaging was acquired with a Leica TCS SP8 system (Leica Microsystems, Germany) equipped with optimized emission/excitation filters for FITC (488/525 nm), Cy3 (552/570 nm), and DAPI (358/461 nm).

### Lipid droplet and lysosome measurements

HepG2 cells were plated in glass-bottom confocal dishes and cultured overnight for adherence. A high-lipid cellular model was induced by FFA treatment, followed by 24-h exposure to CA alone or CA combined with 3-MA. Post-treatment, cells were stained with either Lyso-Tracker Red or LD540 fluorescent probes at 37 °C for 30 min, washed twice with PBS, and imaged using an inverted fluorescence microscope (Nikon, Japan).

### Protein extraction, nucleus and cytoplasm separation, and Western blotting

Proteins were extracted from mouse liver tissues and HepG2 cells using the CytoBuster Protein Extraction Reagent (Sigma-Aldrich) and subsequently quantified using the Bradford Protein Assay Kit (Bio-Rad). Western blotting (WB) was performed with the following antibodies: GAPDH (dilution 1:5000), SIRT1 (dilution 1:1000), FOXO1(dilution 1:1000), AC-FOXO1 (dilution 1:1000), Atg7 (dilution 1:1000), LC3Ⅱ(dilution 1:1000), and p62 (dilution 1:1000). Protein samples were separated using SDS–PAGE (10% gel) and electrotransferred onto PVDF membranes. Membranes were blocked with protein-free buffer (4 h, room temperature) before sequential incubation with primary antibodies (4 °C, overnight) and HRP-conjugated secondary antibodies (45 min, RT). Immunoblot signals were detected using an enhanced chemiluminescence detection system (SQ203L; Epizyme), with band quantification performed in Image-Pro Plus (v6.0). Protein expression was normalized against GAPDH as an internal control across three biological replicates.

### Gene expression analysis and RT-qPCR

Total RNA was isolated using the FastPure® Cell/Tissue Total RNA Isolation Kit V2 (Vazyme Biotech Co., Ltd., Nanjing, China), following the manufacturer’s instructions. RNA integrity was verified by spectrophotometry prior to reverse transcription with reverse transcription enzyme (Yeasen, Shanghai, China). Quantitative PCR amplifications were conducted in triplicate using 2× SYBR Green qPCR Master Mix (Vazyme) on the ABI 7500 Real-Time PCR System (Applied Biosystems). Gene expression quantification employed the comparative 2^−ΔΔCT^ quantification method, normalized against GAPDH as an endogenous control. Primer sequences are detailed in Supplementary Tables 1 and 2.

### shRNA-mediated FOXO1 knockdown

To knockdown FOXO1 expression in HepG2 cells, the shRNA sequence (CCGGCCCTGTATCAACTGCTAAACTCGAGTTTCAGCATTGATAACAGGCTTTTTG) was cloned into the shRNA insertion site of the pLKO.1-EGFP-puro vector. Lentiviral particles carrying the shRNA were produced by co-transfecting 293T cells with the psPAX2 and pMD2.G plasmids along with the recombinant vector. Forty-eight hours post-transfection, the viral supernatant was harvested and filtered through a 0.45 μm membrane. HepG2 cells were infected with this lentivirus for 48 h, followed by selection with puromycin (2.5 μg mL⁻¹) to obtain stably infected cells.

### DNA isolation and 16S rRNA sequencing

Bacterial genomic DNA was isolated from fecal specimens using the QIAamp® DNA Stool Mini Kit (QIAGEN, Hilden, Germany). DNA quality was assessed by both 1% agarose gel electrophoresis and NanoDrop One spectrophotometry (Thermo Fisher Scientific, Waltham, MA, USA), with aliquots stored at −80 °C. The V3–V4 hypervariable regions of 16S rRNA genes were amplified using Q5® Hot Start High-Fidelity DNA Polymerase (NEB, Ipswich, MA, USA) with modified 341F/805R primers (Integrated DNA Technologies). PCR products were indexed with Illumina paired-end adapters using ~2–5 μL template DNA per reaction prior to sequencing. The gut microbiota composition and diversity in mice were characterized by Illumina HiSeq 2500 sequencing (I-Sanger, Shanghai, China). Raw sequencing data were processed using the QIIME2 pipeline (version 2020.6). After quality filtering, sequences were clustered into operational taxonomic units (OTUs) at 97% similarity threshold using an open-reference OTU-picking strategy against the Greengenes database (v13_8). QIME was used for the principal coordinates analysis depending on the unweighted unifrac distances, and inter-group comparisons were conducted using Wilcoxon rank-sum tests.

### Fecal microbiota transplantation (FMT)

Eighteen mice were randomly assigned to the NC group (*n* = 6) and HFD group (*n* = 12). After 8 weeks of HFD induction, the HFD group mice were further randomized into the model group (*n* = 6) and the FMT group (*n* = 6). Mice in the FMT group received a one-week combination antibiotic treatment containing 100 g/L neomycin, 100 g/L penicillin, 50 g/L vancomycin, and 100 g/L metronidazole to deplete endogenous gut microbiota. Starting in week 9, fresh fecal samples from CA-H mice were used as donor material. These samples were homogenized in anaerobic PBS at a 1:10 (w/v) ratio. The suspension was vortexed and shaken on a rotary shaker for three minutes to ensure thorough microbial mixing, then filtered through a sterile stainless-steel mesh sieve with a 0.25 mm pore size. The FMT group then received daily oral gavage of 0.2 mL of the fecal suspension, while the NC and model groups received an equivalent volume of distilled water via gavage. This intervention lasted for six weeks.

### SCFAs quantification

Fecal samples were homogenized in 0.1 M HCl, centrifuged, and extracted with diethyl ether. After evaporation, the residue was derivatized with MTBSTFA/TBDMCS at 60 °C for 30 min. GC–MS analysis used a DB-FFAP column with temperature programming (50 °C → 200 °C at 10 °C/min). Helium carrier gas (1.0 mL/min) and electron impact ionization (70 eV) were employed. Quantification was performed via selected ion monitoring (*m*/*z* 73, 87, 101 for acetic, propionic, and butyric acids, respectively) using calibration curves (*R*² > 0.995). The method showed good precision (RSD < 5%) and recoveries (90–110%), with detection limits in the low μg/mL range.

### Statistical analysis

All statistical analyses were conducted using ‌GraphPad Prism 9.0 software‌ (GraphPad Software, San Diego, CA, USA). Specifically, all figures except Fig. 12 were generated using GraphPad Prism 9.0 software (GraphPad Software, San Diego, CA, USA) and typeset with Adobe Illustrator (‌Adobe, San Jose, CA, USA). Figure 12 was created using BioRender (https://www.biorender.com/, agreement number: BU29BP9GV3) and Adobe Illustrator (Adobe, San Jose, CA, USA). Sample sizes for the in vivo experiments are specified in the corresponding figure legends. Depending on the data characteristics, appropriate statistical tests were applied: ‌unpaired two-tailed Student’s *t*-test, ‌one-way ANOVA, or ‌nonparametric rank sum test, with statistical significance set at ‌*P* < 0.05. Additionally, ‌Spearman’s correlation analysis‌ was performed using ‌OmicStudio‌ (https://www.omicstudio.cn). Significance levels were denoted as follows: ^*#*^*P* < 0.05, ^##^*P* < 0.01, *P < 0.05, **P < 0.01.

## Supplementary information

Supplementary information

## Data Availability

The datasets generated and/or analyzed during the current study are not publicly available due to the fact that related research is still in progress, but are available from the corresponding author on reasonable request. The raw data and metadata are maintained on the Majorbio cloud platform (www.majorbio.com), where the omics sequencing was performed.
