# Supplementary material for: Cinnamaldehyde mitigates MASLD through SIRT1/FOXO1-induced autophagy and synergistic gut microbiota modulation
Source: NPJ Sci Food. 2026 Apr 9;10:183. doi: 10.1038/s41538-026-00815-6 (PMC13249847; doi:10.1038/s41538-026-00815-6)
Supplement: Supplementary file 1 — Supplementary information [file 41538_2026_815_MOESM1_ESM.docx]

Supplementary Materials for

**Cinnamaldehyde mitigates MAFLD through SIRT1/FOXO1-induced autophagy and synergistic gut microbiota modulation**

Xiaoran Wang^a^, Yagang Song^c^, Wenyu Zhao^d^, Yuting Liu^e^, Yiping Fu^c^, Yu Zhang^c^, Quanyou Zhao^e^, Mingsan Miao^c^, Wenxia Zhao^a^, Xianbo Wang^f#^, Zhanzhan Li^b#^

*^a^Department of Digestive Diseases, The First Affiliated Hospital of Henan University of Chinese Medicine, Zhengzhou 450046, China*

*^b^First Clinical Medical College, Henan University of Chinese Medicine, Zhengzhou 450046, China*

*^c^Academy of Chinese Medicine Sciences, Henan University of Chinese Medicine, Zhengzhou 450046, China*

*^d^School of Rehabilitation Sciences, Henan University of Chinese Medicine, Zhengzhou 450046, China*

*^e^Pharmacy College, Henan University of Chinese Medicine, Zhengzhou 450046, China*

*^f^Center for Integrative Medicine, Beijing Ditan Hospital Capital Medical University, Beijing 100015, China*

**#** Correspondence author:

Zhanzhan Li: wind_lz@hactcm.edu.cn; Academy of Chinese Medicine Sciences, Henan University of Chinese Medicine, No. 156 Jinshuidong Road, Zhengzhou 450046, China.

Xianbo Wang: wwwzp2020@163.com; Center for Integrative Medicine, Beijing Ditan Hospital Capital Medical University, No. 8 Jingshun East Street,Beijing 100015, China.

**Supplementary tables and figures**

**Supplementary Table 1. The primers used in qPCR analysis. （mice）**

| **Target** | **Sequence (5’-3’)** |
| --- | --- |
| GAPDH | Forward5’-CCTCGTCCCGTAGACAAAATG-3’  Reverse5’-TGAGGTCAATGAAGGGGTCGT-3’ |
| SREBP-1c | Forward5’-GACATGCTCCAGCTCATCAACA-3’  Reverse5’-GACACGGACGGGTACATCTTTA-3’ |
| FAS | Forward5’-CTGCCTCTGGTGCTTGCT-3’  Reverse5’-ACCCGCCTCCTCAGCTTT-3’ |
| ACC1 | Forward5’-TTTGTTTGGTCGTGACTGCTCTG-3’  Reverse5’-AGGATGTTCAACCTGTAGCCGAG-3’ |
| PPARα | Forward5’-TTTCACAAGTGCCTGTCTGTCG-3’  Reverse5’-TCTTCAGGTAGGCTTCGTGGAT-3’ |
| CPT-1 | Forward5’-GCCTCTATGTGGTGTCCAAGTATC-3’  Reverse5’-CACCATAGCCGTCATCAGCAA-3’ |
| TFEB | Forward5’-AATCCCACATCCTACCATCTGC-3’  Reverse5’-GCAGCAAACTTGTTCCCATAGG-3’ |
| SIRT1 | Forward5’-ACATCTCATGATTGGCACCGAT-3’  Reverse5’-AGCGTCATATCATCCAGCTCAG-3’ |
| FoxO1 | Forward5’-TACTTCAAGGATAAGGGCGACAG-3’  Reverse5’-TGCACTCGAATAAACTTGCTGTG-3’ |

**Supplementary Table 2. The primers used in qPCR analysis. （HepG2 cells）**

| **Target** | **Sequence (5’-3’)** |
| --- | --- |
| GAPDH | Forward5’-GGAAGCTTGTCATCAATGGAAATC-3’  Reverse5’-TGATGACCCTTTTGGCTCCC-3’ |
| SREBP-1c | Forward5’-TCTGGAGGCATCGCAAGC-3’  Reverse5’-AGCAGGTGACGGATGAGGTT-3’ |
| FAS | Forward5’-CATCTGGACCCTCCTACCTCTG-3’  Reverse5’-CTGTGTACTCCTTCCCTTCTTGG-3’ |
| ACC1 | Forward5’-TGCGGTCTATCCGTAGGTGGT-3’  Reverse5’-CATAGTTGTTGTTGTTTGGTCCTCC-3’ |
| PPARα | Forward5’-CCTCGGTGACTTATCCTGTGGT-3’  Reverse5’-GACATCCCGACAGAAAGGCAC-3’ |
| CPT-1 | Forward5’-GAAGTTGTTCAAGTTGGCGTCT-3’  Reverse5’-ATGTACGACACACCATAGCCGT-3’ |
| TFEB | Forward5’-AATCCCACATCCTACCATCTGC-3’  Reverse5’-GCAGCAAACTTGTTCCCATAGG-3’ |
| SIRT1 | Forward5’-ATTCCAGCCATCTCTCTGTCAC-3’  Reverse5’-TGTTGCAAAGGAACCATGACAC-3’ |
| FoxO1 | Forward5’-TACTTCAAGGATAAGGGTGACAGC-3’  Reverse5’-TTTTCCAGTTCCTTCATTCTGCAC-3’ |

**Supplementary Figure 1**


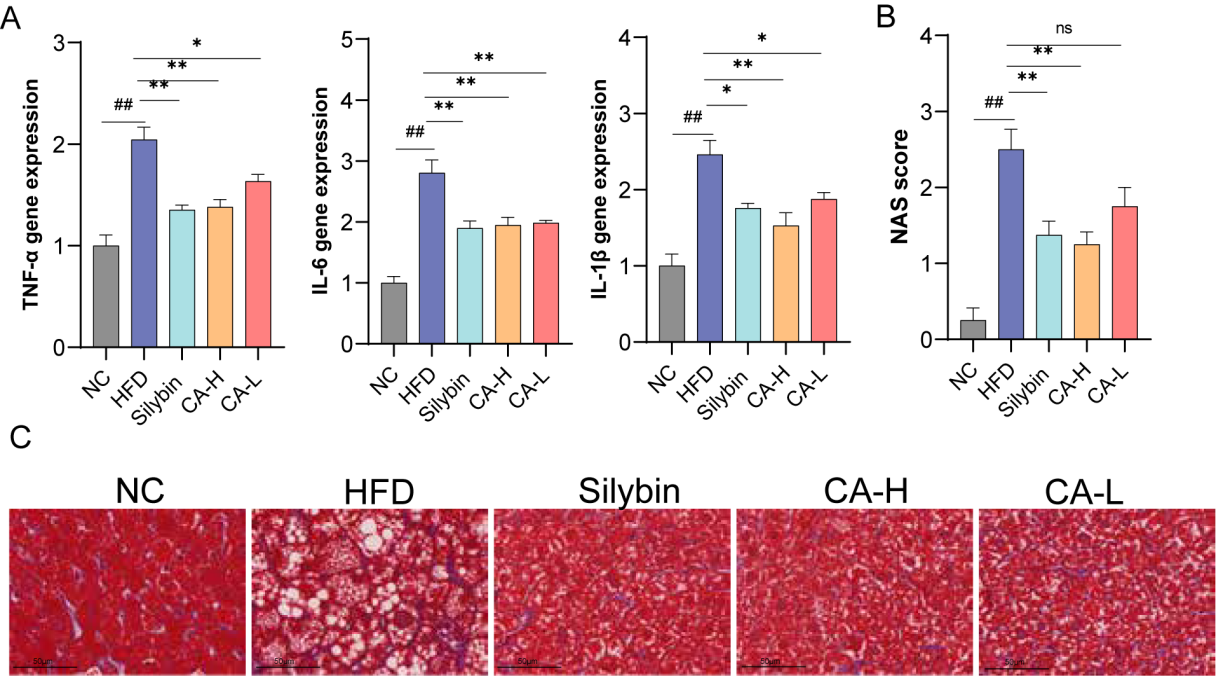


Figure S1. CA ameliorates hepatic inflammation and pathological changes in HFD-induced mice. (A) Hepatic mRNA expression of TNF-α, IL-6, and IL-1β determined by RT-qPCR. (B) NAFLD activity score (NAS) based on histological evaluation. (C) Representative photomicrographs of Masson’s trichrome staining of liver sections. Blue staining indicates collagen deposition, and red staining indicates cytoplasm (Scale bar = 50 μm). Data are shown as the means ± SEMs (n=8). Compared with the NC , ^#^*P* < 0.05, *^##^P* < 0.01; compared with the HFD group, **P* < 0.05, ***P* < 0.01

**Supplementary Figure 2**

**
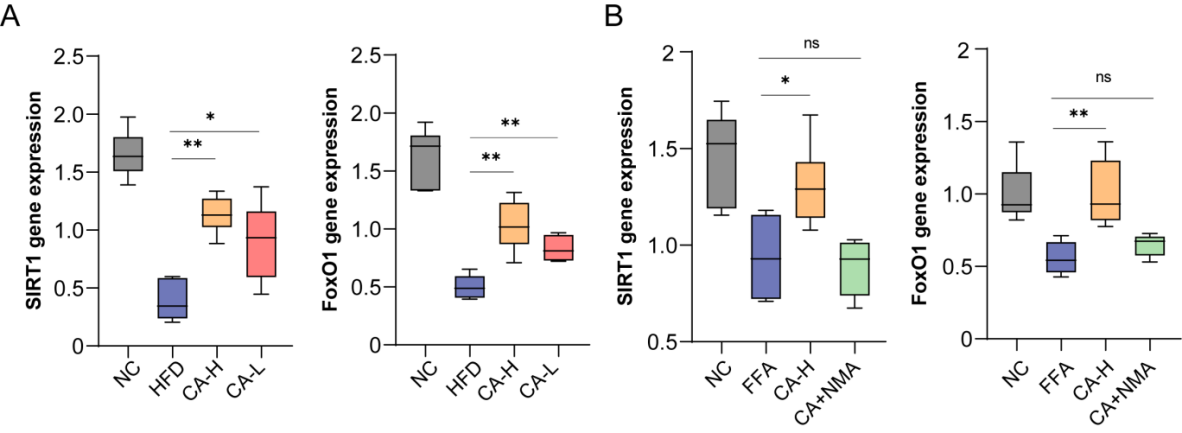
**

Fig. S2 The mRNA expression levels of SIRT1 and FOXO1. (A) The mRNA expression levels of SIRT1 and FOXO1 in liver tissue (n=6). (B) The mRNA expression levels of SIRT1 and FOXO1 in FFA-induced HepG2 cells. Data are shown as the means±SEMs (n=6). Compared with the HFD or FFA group, **P* < 0.05, ***P* < 0.01.

**Supplementary Figure 3**


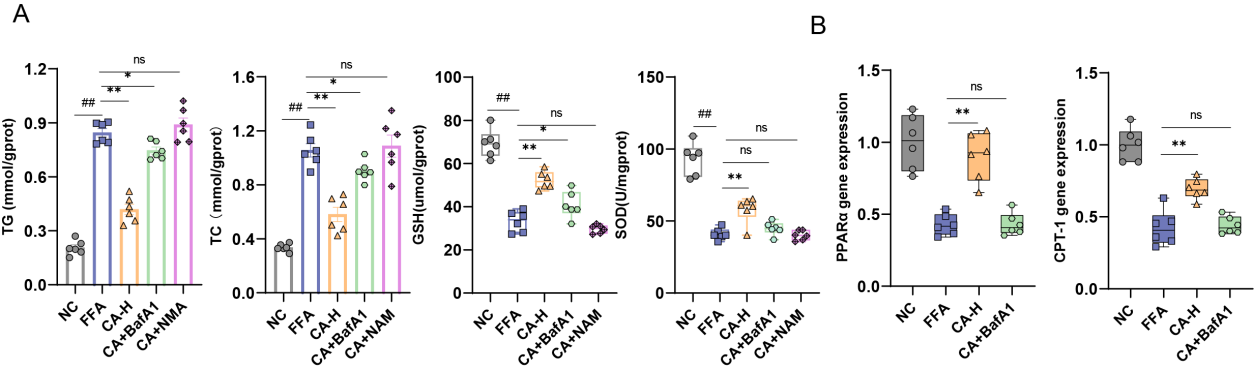


Fig. S3 Treatment with BafA1 inhibits CA’ s effect in HepG2 cells. (A) Intracellular TG 、TC、GSH and SOD levels. (B) The mRNA expression levels of fatty acid oxidation genes (PPARα, CPT-1). Data are shown as the means ± SEMs (n=6). Compared with the NC group, ^#^*P* < 0.05, ^##^*P* < 0.01; compared with the FFA group, **P* < 0.05, ***P* < 0.01.

**Supplementary Figure 4**


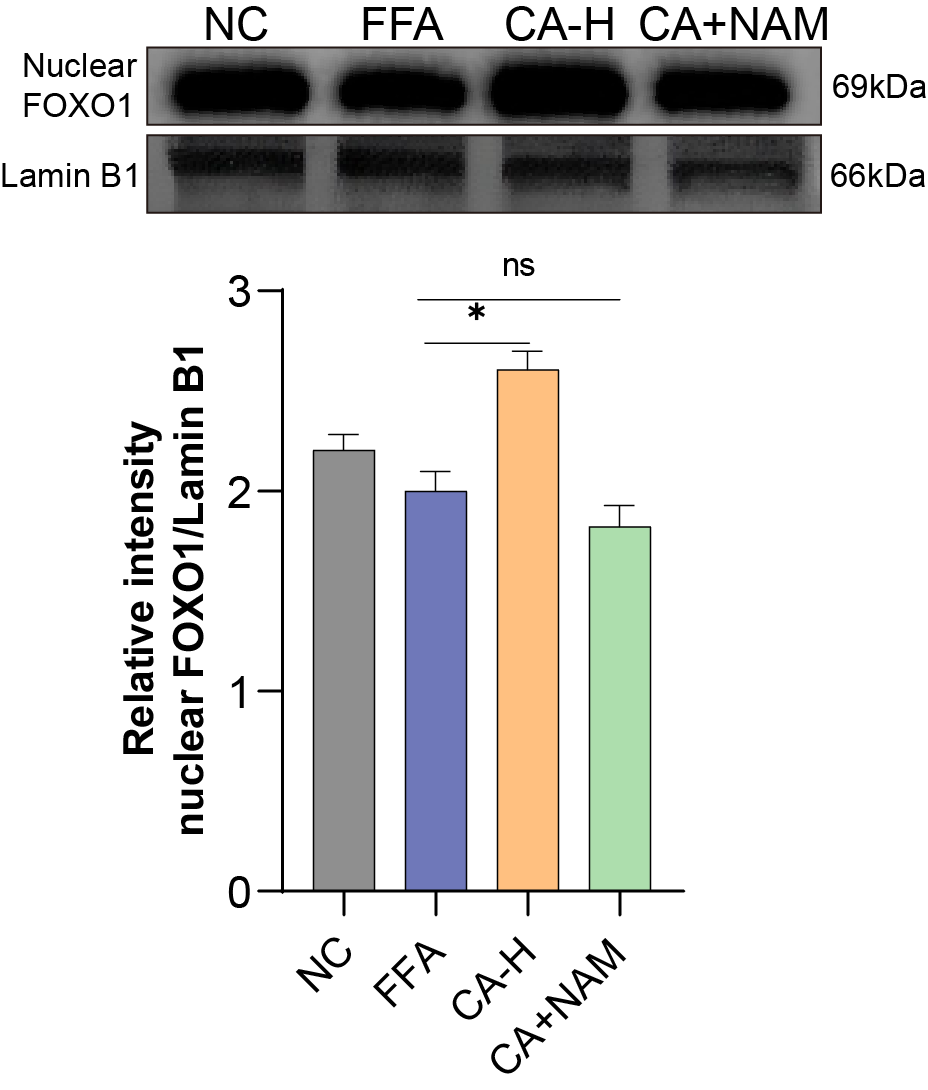


Fig S4 Nuclear FOXO1 expression. Data are shown as the means ± SEMs (n=3). Compared with the FFA group, **P* < 0.05, ***P* < 0.01.

**Supplementary Figure 5**


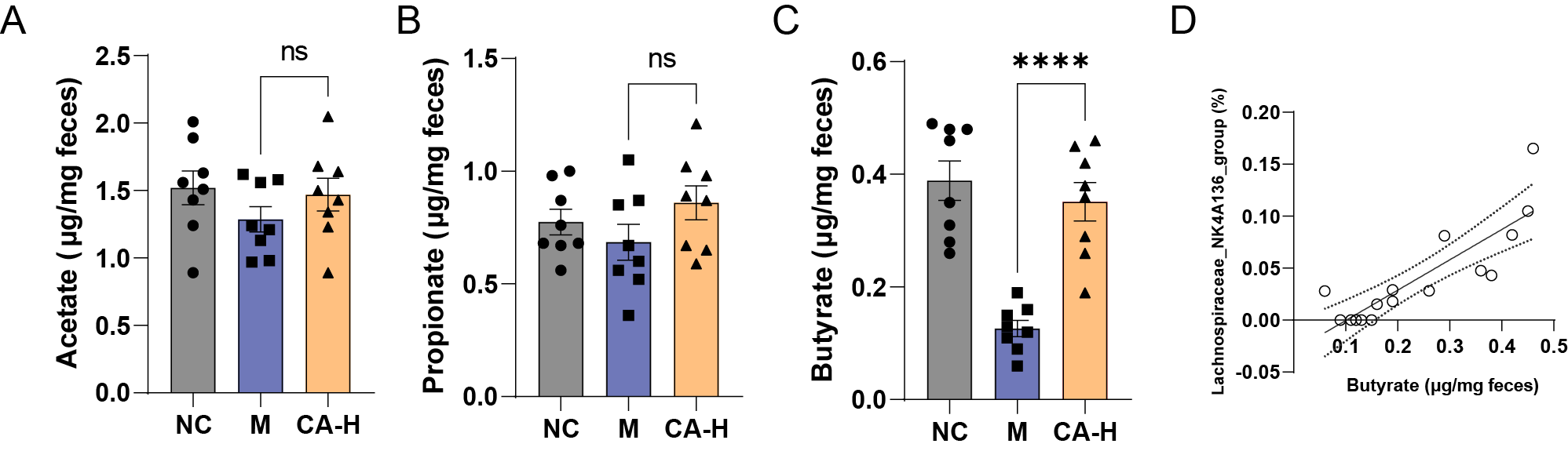


Fig. S5 CA modulates fatty acids‌. ‌(A) Acetic acid, (B) propionic acid, and (C) butyric acid levels in mouse feces.‌ ‌(D) Correlation analysis between fecal butyric acid content and *Lachnospiraceae_ NK4A136_group* abundance in HFD and CA-H group mice. Data are shown as the means ± SEMs (n=8). Compared with the HFD group, **P* < 0.05, ***P* < 0.01.

**Supplementary Figure 6**

**Fig. 5A** **Uncropped images for gels and blots.**

Original scan images are shown for the indicated figure panels, and the red boxes representthe cropped areas corresponding to the bands presented in the text . The blue squares denote two additional replicates, making a total of three replicates.Molecular weight markers and the antibodies used are indicated.

**
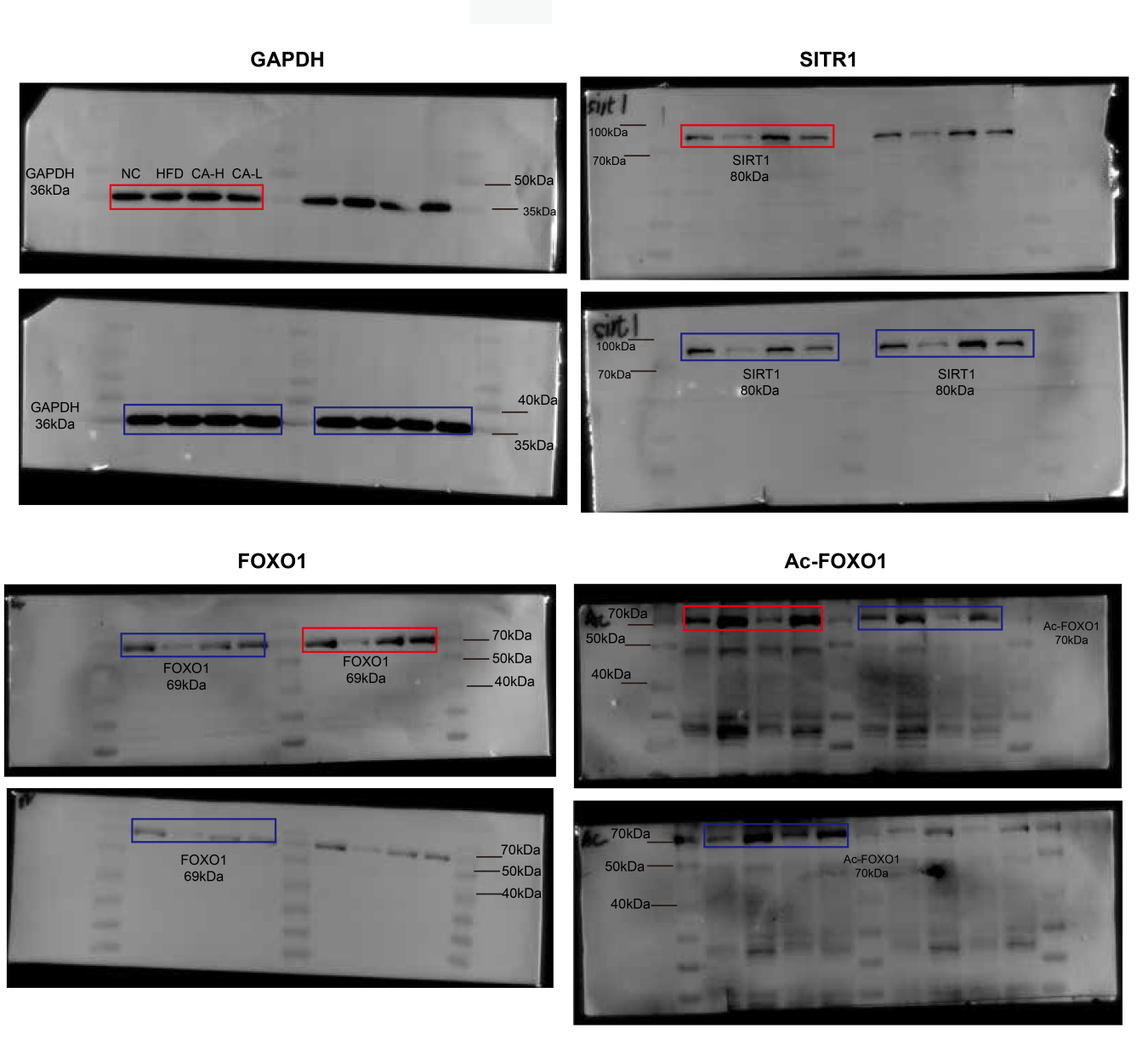
**

**Supplementary Figure 7**

**Fig. 5D** **Uncropped images for gels and blots.**

Original scan images are shown for the indicated figure panels, and the red boxes representthe cropped areas corresponding to the bands presented in the text. The blue squares denote two additional replicates, making a total of three replicates.Molecular weight markers and the antibodies used are indicated.


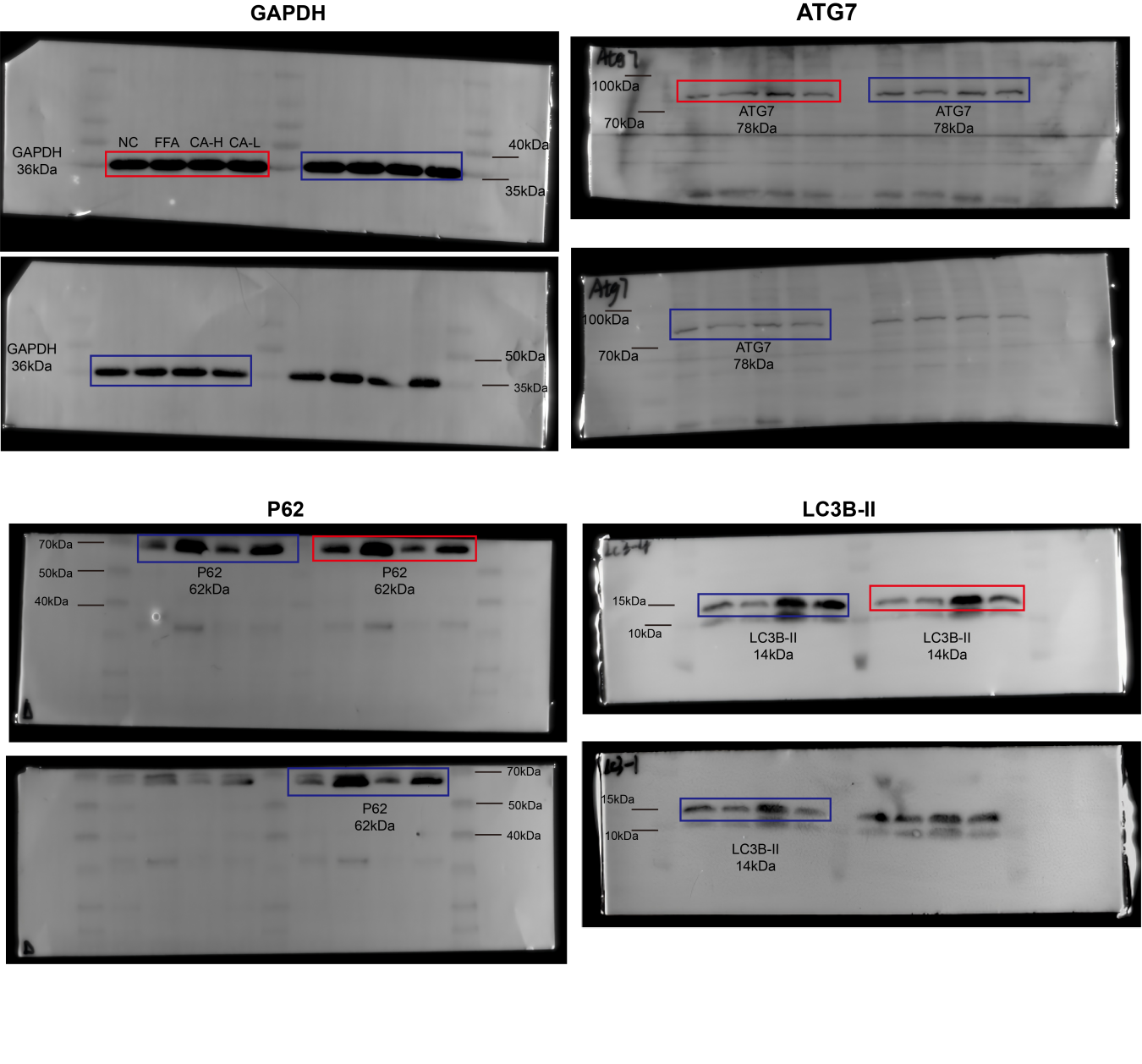


**Supplementary Figure 8**

**Fig. 7A** **Uncropped images for gels and blots.**

Original scan images are shown for the indicated figure panels, and the red boxes representthe cropped areas corresponding to the bands presented in the text. The blue squares denote two additional replicates, making a total of three replicates. Molecular weight markers and the antibodies used are indicated.


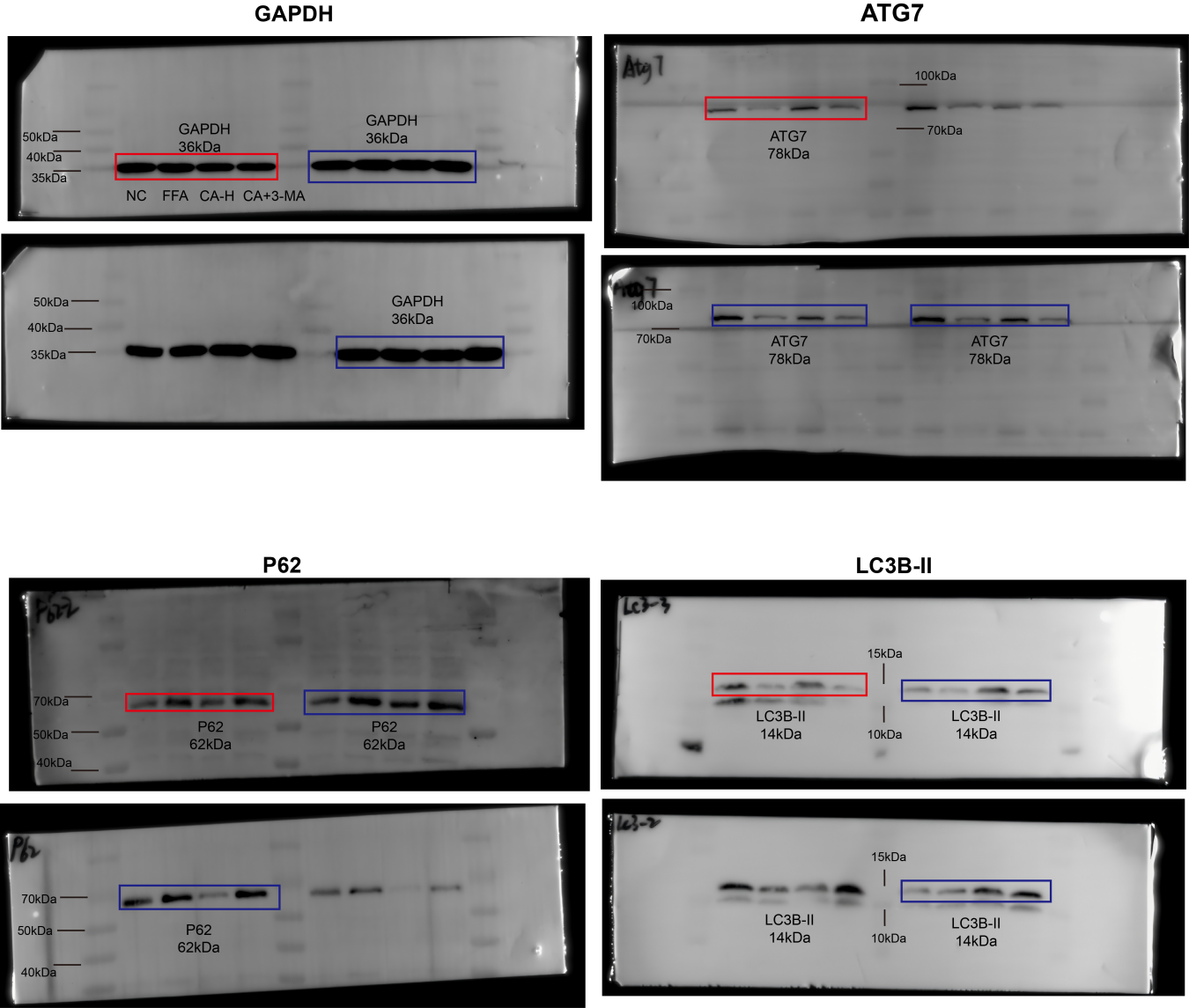


**Supplementary Figure 9**

**Fig. 8A** **Uncropped images for gels and blots.**

Original scan images are shown for the indicated figure panels, and the red boxes representthe cropped areas corresponding to the bands presented in the text . The blue squares denote two additional replicates, making a total of three replicates.Molecular weight markers and the antibodies used are indicated.


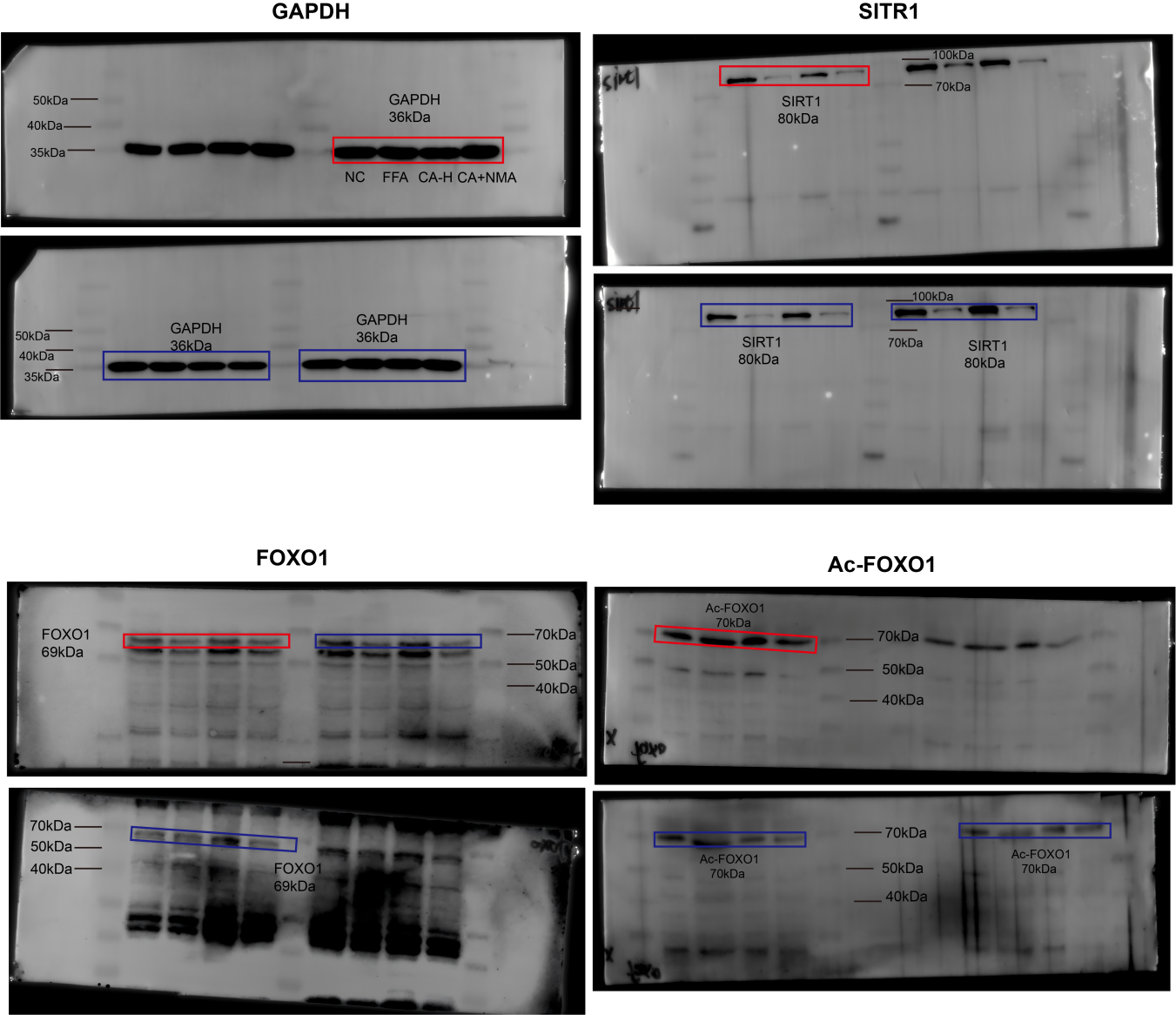


**Supplementary Figure 10**

**Fig 9A Uncropped images for gels and blots.**

Original scan images are shown for the indicated figure panels, and the red boxes representthe cropped areas. Molecular weight markers and the antibodies used are indicated.

**
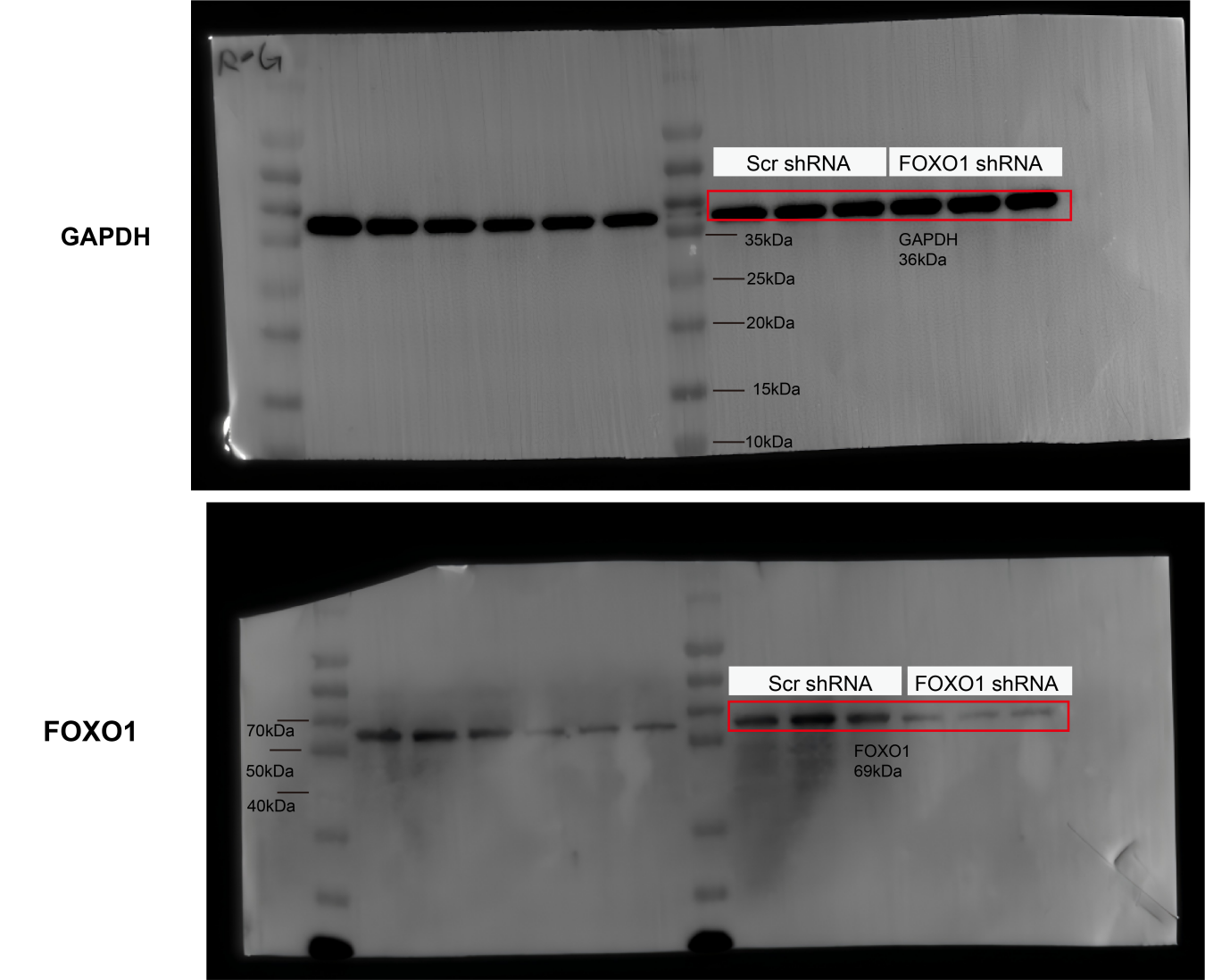
**

**Supplementary Figure 11**

**Fig. S4 Uncropped images for gels and blots.**

Original scan images are shown for the indicated figure panels, and the red boxes representthe cropped areas corresponding to the bands presented in the text. The blue squares denote two additional replicates, making a total of three replicates.Molecular weight markers and the antibodies used are indicated.

**
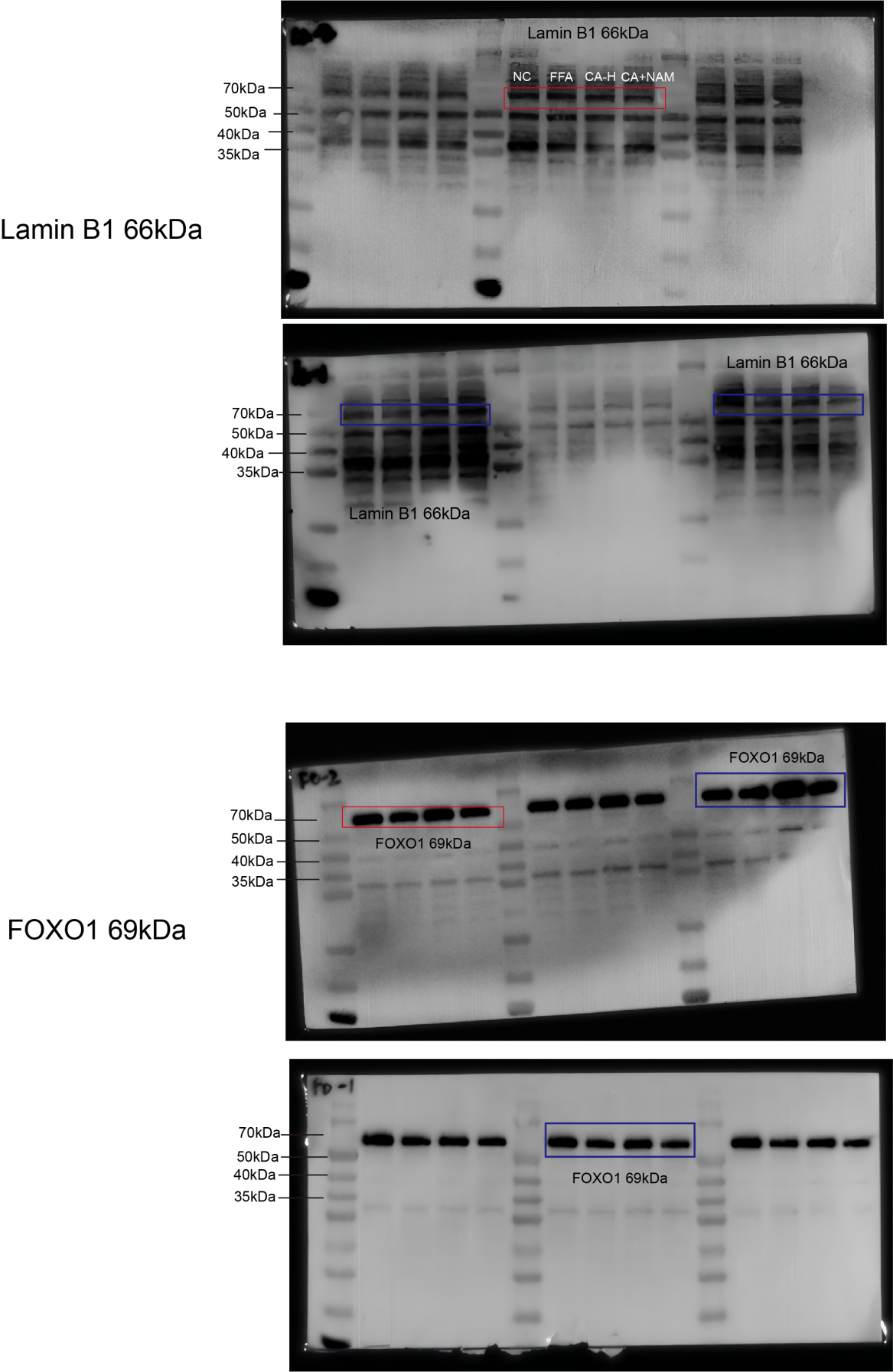
**
